# Toroidal displacement of *Klebsiella pneumoniae* by *Pseudomonas aeruginosa* is a unique mechanism to avoid competition for iron

**DOI:** 10.1128/mbio.01149-25

**Published:** 2025-06-11

**Authors:** Diana Pradhan, Ajay Tanwar, Joshua Wong, Srividhya Parthasarathi, Gad M. Frankel, Varsha Singh

**Affiliations:** 1 Department of Developmental Biology & Genetics, Indian Institute of Science 29120, Bangalore, India; 2 Division of Moleculer Microbiology, University of Dundee, Dundee, United Kingdom; 3 Department of Life Sciences, Imperial College 98455, London, United Kingdom; 4 Department of Physics, Indian Institute of Science 534309, Bangalore, India; Florida International University, Miami, Florida, USA; Columbia University, New York, New York, USA

**Keywords:** *Pseudomonas aeruginosa*, *Klebsiella pneumoniae*, competition, iron, rhamnolipid

## Abstract

Competition for resources is one of the major drivers for evolution and retention of new traits in microbial communities. Quorum sensing-dependent traits of the opportunistic human pathogen *Pseudomonas aeruginosa* allow it to survive and thrive in the presence of other microbes. Here, we report a unique mechanism that *P. aeruginosa* employs specifically against *Klebsiella pneumoniae* to displace it on solid surfaces. Interestingly, *P. aeruginosa* employs neither proteases nor toxic secondary metabolites against *K. pneumoniae*. Rhamnolipid biosurfactant under the control of the RhlR quorum sensing system appears to be the primary factor required to displace *Klebsiella* effectively. Under conditions of iron limitation, both bacteria produce iron-scavenging molecules, while *P. aeruginosa* also produces rhamnolipid biosurfactant, allowing it to push *Klebsiella* cells away from the substratum. Our study describes a unique quorum and iron-responsive mechanism in *P. aeruginosa* to support its own growth during resource competition on a solid surface.

**IMPORTANCE:**

Competition is common among organisms in nature. Bacteria have been known to deal with their neighbors using toxins and enzymes that kill or disable the latter. Here, we describe a simple, detergent-mediated mechanism that a common bacterium employs against its neighbor to push them away. This only happens in a time of scarcity of iron, an element essential for life, big and small.

## INTRODUCTION

In most natural settings, microbes are generally known to occur as multispecies consortia, including in pathophysiological conditions like cystic fibrosis, pneumonia, urinary tract infection, and diabetic foot ulcer. Microbes with diverse metabolic capacities can cooperate and coexist in a consortium. However, pathogens occupying a common niche might compete for macro or micronutrients, resulting in altered virulence and persistence of one or more pathogens. These interactions are seen inside a host or in the environment.

Microbial responses can be profoundly altered by the presence of another microbe. Interspecies competition enhances the production of virulence factors in *Pseudomonas aeruginosa* as seen in the mixed biofilms of *P. aeruginosa* and *Candida albicans* (1). The presence of *Staphylococcus aureus* has been shown to cause differential expression of hundreds of genes in *P. aeruginosa* compared to its monoculture (2). Mono versus dual infection of the animal host also reflects an alteration in pathogenesis and disease progression (3). Most human infections are polymicrobial (4). Clinical outcomes for coinfections involving two or more pathogens are more severe than infection with a single pathogen in some cases (5, 6). These include pneumonia (7, 8), wound infections (9, 10), diabetic foot ulcer (11, 12), and intestinal inflammation (13). Therefore, there is a need to understand pathogenesis influenced by inter-microbial interactions.

Bacterial pneumonia is often characterized by the presence of many species of bacteria. *P. aeruginosa*, *S. aureus*, *K. pneumoniae*, and *Acinetobacter baumannii* are some of the commonly occurring antibiotic-resistant pathogens in hospital-acquired pneumonia (14). *P. aeruginosa* and *K. pneumoniae* are known to share a common niche in lung pathologies, in sepsis, and in chronic wound infections (12, 15, 16). Both species can coexist as a stable dual-species biofilm (17). Under nutrient-replete conditions, LasB protease of *P. aeruginosa* is necessary for the dispersal of the *in vitro* biofilm of *K. pneumoniae* (18). Intriguingly, *P. aeruginosa* growth is inhibited by *K. pneumoniae* in rich media, whereas its growth is supported by *K. pneumoniae* under biotin-limiting conditions via an unknown mechanism (15). These reports show that the outcome of interactions between these two bacteria is highly contextual.

Pathogens with specific virulence factors have an advantage over neighboring bacteria. These factors include toxic molecules, such as phenazines and hydrogen cyanide produced by *P. aeruginosa* (19, 20). Some bacteria also utilize specific surfactants to disperse from biofilm or use surfactant-aided motility, such as swarming, to utilize resources in a given area (21–23).

In this study, we examined environmental and molecular determinants of interactions between *P. aeruginosa* and *K. pneumoniae* under nutrient-limiting conditions. We established a coculture assay on agar surface and found that *P. aeruginosa* and *K. pneumoniae* coexist on complex, nutrient-rich media. On minimal media, *P. aeruginosa* rapidly pushes away *K. pneumoniae*. The displacement of *K. pneumoniae* by *P. aeruginosa* is independent of all its virulence factors, except rhamnolipids. We find that the pushing behavior of *P. aeruginosa* can be suppressed by the supplementation of iron. On minimal media, *P. aeruginosa* and *K. pneumoniae* produce several siderophores, each setting off competition for iron. This results in surfactant production in *P. aeruginosa*, allowing it to push away nonmotile *K. pneumoniae* from the surface. Our study has uncovered an additional function of the surfactant that bacteria can utilize to push competitors away.

## RESULTS

### Interaction of *P. aeruginosa* with *K. pneumoniae* is media dependent

**Fig 1:**
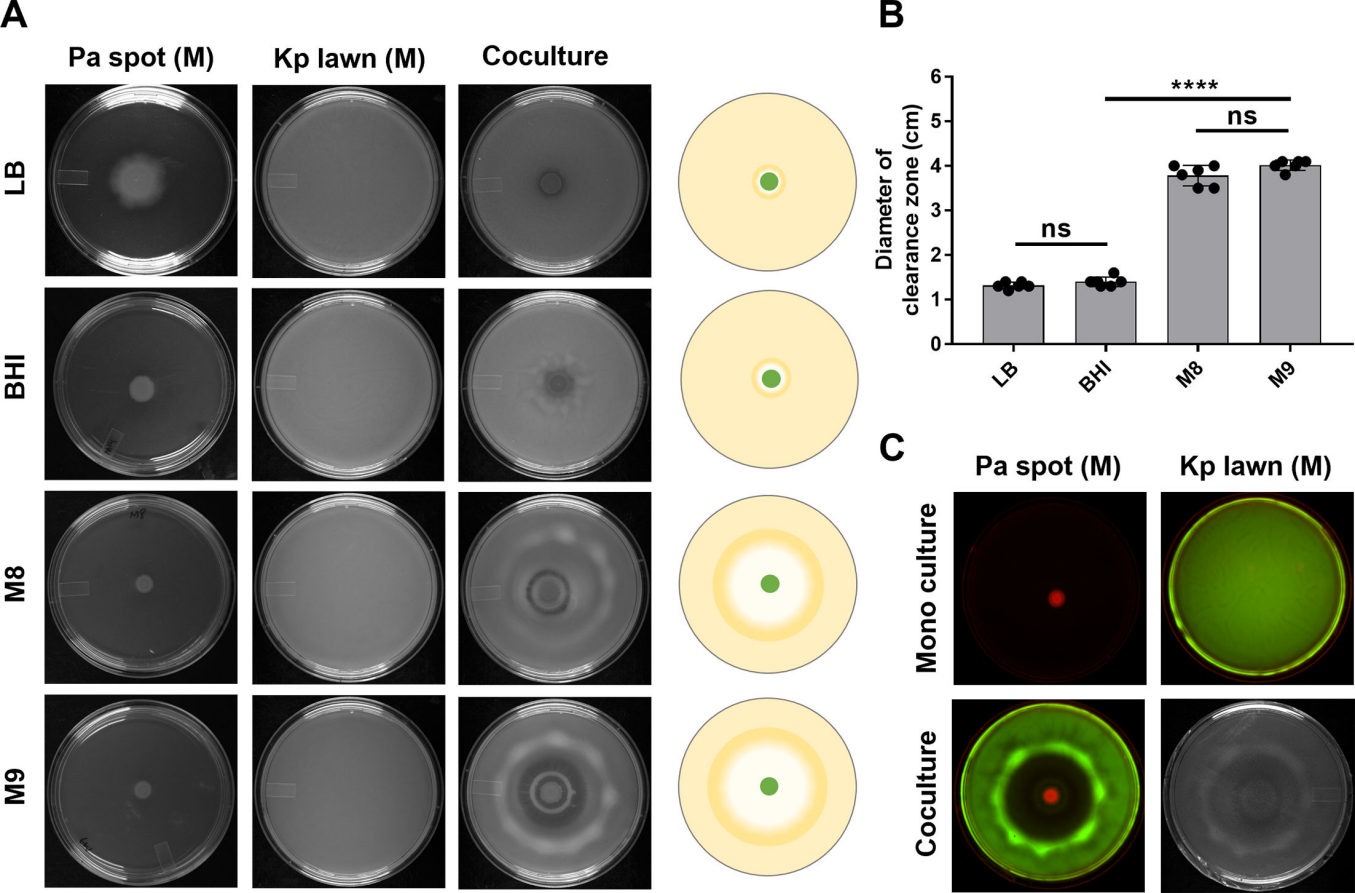
Interaction of *Pseudomonas aeruginosa* and *Klebsiella pneumoniae* is media-dependent. (**A**) *P. aeruginosa* (Pa) and *K. pneumoniae* (Kp) monocultures (M) and cocultures (C) on LB, BHI, M8, M9 media solidified with 1% agar in 90 mm petri plate. A schematic representation of the spot-on-lawn interaction assay is shown on the right side. (**B**) Quantification of the clearance zone on LB, BHI, M8, and M9 plates. One-way analysis of variance, followed by Tukey’s *post-hoc* test was used for the analysis of significance (ns, nonsignificant; ****, *P*  ≤  0.0001). (**C**) Interaction of *P. aeruginosa* expressing mCherry and *K. pneumoniae* expressing GFP on M9 plate. *n* represents the number of biological replicates. Error bars indicate SEM.

To understand the rules of engagement between *P. aeruginosa* (Pa) and *K. pneumoniae* (Kp), we examined their interaction on solid media with varying nutritional content. We studied mono and coculture of Pa and Kp 24 h after plating them on lysogeny broth (LB), brain heart infusion (BHI), M9 minimal medium, or M8 minimal medium plates solidified with 1% agar. For Kp monoculture, KPPR1 was spread on the entire surface of a 90 mm plate, creating a lawn, while for Pa monoculture, 5 µL of an overnight culture of PA14 was spotted at the center of another 90 mm plate. For coculture experiments, we developed a spot-on-lawn interaction assay. The center of a lawn of Kp was spotted with 5 µL of an overnight culture of Pa (Fig. 1A). Kp exhibited profuse growth as a monoculture lawn in 24 h at 37°C. Pa spot, as monoculture, grew in the center only up to a 4 mm circle under the same conditions. On the spot-on-lawn interaction plates, Pa spot grew to a diameter of ~7 mm, while a much larger clearance zone emerged around the Pa spot on M9 and M8 plates (Fig. 1A). In contrast, no clearance was observed around Pa on LB and BHI coculture plates (Fig. 1A). It is worth noting that other traits of Pa are also dependent on growth media. For example, Pa displays swarming, a rhamnolipid-dependent collective motility, on M8 and M9 media but not on rich media, BHI and LB (21, 24, 25). Quantification of the clearance zone also indicates that M8 and M9 media support the emergence of a clearance zone on Pa-Kp interaction plates (Fig. 1B). The clearance of Kp from the area surrounding Pa was also visualized using GFP-expressing Kp and mCherry-expressing Pa on M9 plates (Fig. 1C).

To understand whether clearance emerged as a response of Pa or of Kp, we also examined the reciprocal interaction wherein we plated Pa cells on the entire agar surface, followed by spotting Kp cells in the center. In this scenario, no zone of clearance appeared (Fig. S1A). This indicated that the clearance zone emerged because of the action of *P. aeruginosa*, and there is no direct inhibition of *P. aeruginosa* by *K. pneumoniae*.

To understand whether the clearance of Kp is performed explicitly by Pa, we studied the behavior of other bacteria against Kp on M9 agar. We spotted *S. aureus*, *Escherichia coli*, or *Proteus mirabilis* on the lawn of Kp but did not observe any clearance (Fig. S1B), suggesting that the clearance of Kp cells is not a function broadly performed by many bacterial species. We also examined the behavior of Pa against other gram-negative bacteria. As shown in Fig. S2, *P. aeruginosa* did not push away *E. coli*, *P. mirabilis*, *Salmonella enterica*, or *Serratia marcescens* as it did Kp. This experiment suggested that the Pa-Kp interaction is unique to this pair of strains under the specific conditions investigated here, and clearance originates from the action of *P. aeruginosa*. Altogether, our experiments indicate that some factor(s) in the M9 medium promotes the action of Pa against Kp.

### Toroidal displacement of *K. pneumoniae* by *P. aeruginosa* on minimal media

**Fig 2:**
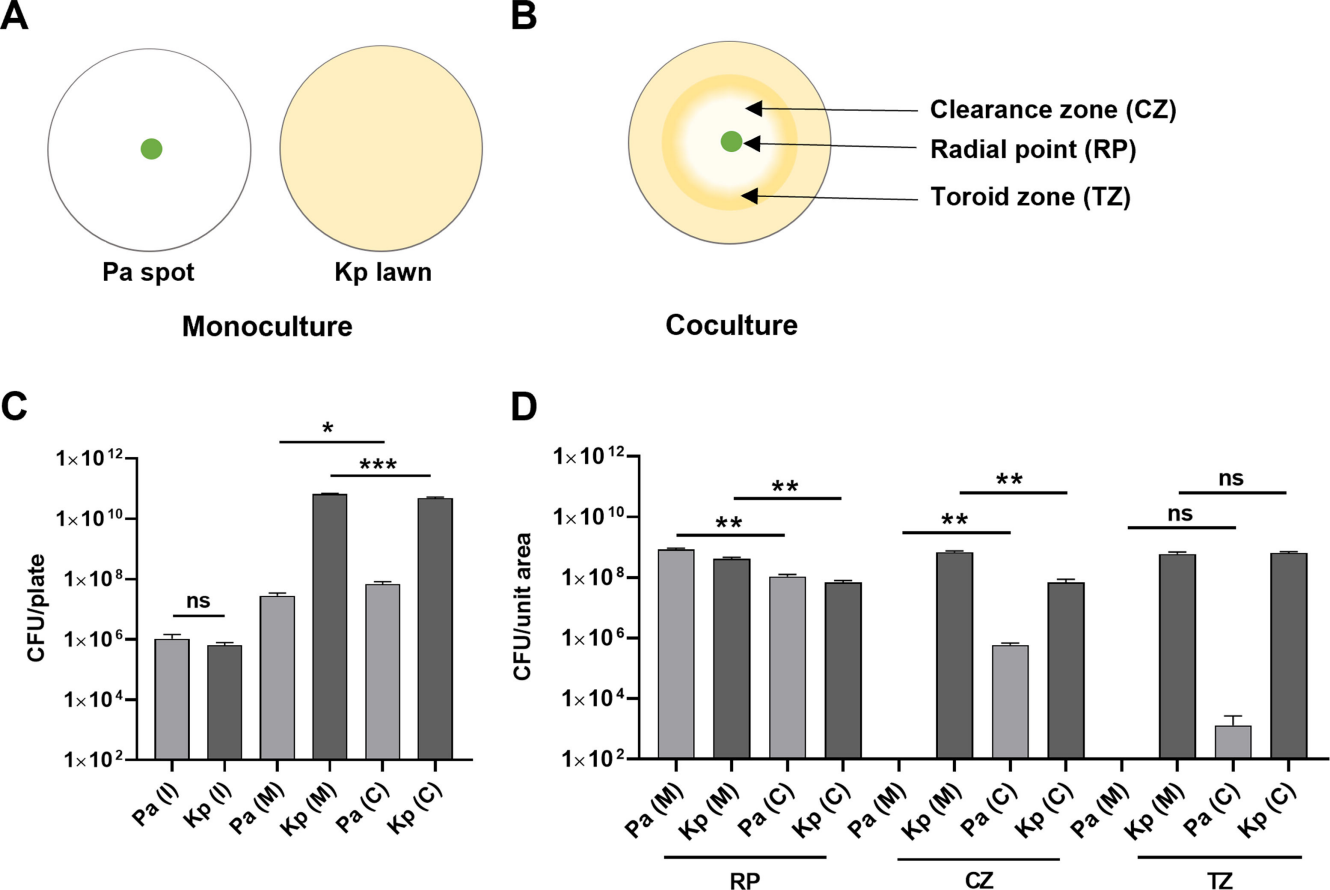
Toroidal displacement of *K. pneumoniae* by *P. aeruginosa* on a solid surface. (**A**) Schematic representation of Pa and Kp monoculture plates. (**B**) Schematic representation of Pa and Kp on M9 coculture plates showing different zones: radial point (RP), clearance zone (CZ), and toroid zone (TZ). (**C**)Enumeration of the total CFU of Pa and Kp from inoculum (I), monoculture (M), and coculture (C) plates. (**D**) Enumeration of CFU of Pa and Kp from different zones of monoculture (M) and coculture (C) spot-on-lawn interaction plates. An unpaired *t*-test was used for the analysis of significance for pairwise comparisons in panels C and D (ns, nonsignificant; *, *P*  ≤  0.05; **, *P*  ≤  0.01). Three to four biological replicates were used for analysis. Error bars indicate SEM.

To better understand the interaction between Pa and Kp, we closely monitored the development of the clearance zone in coculture plates in a time-lapse movie (see Movie S1). We observed a wave emanating from Pa cells present in the center (radial point [RP]) pushing Kp cells outward. This created a clearance zone (CZ) and a mass of material collected in the toroid zone (TZ) (Fig. 2B). This indicated that Kp cells were being actively pushed radially outward to form a doughnut or toroid shape, where Kp cells and possibly other materials accumulated, making a ring-like appearance (Fig. 2B). We term this pushing phenomenon to form a doughnut or toroid shape as “*'*toroidal displacement.”

To understand the effect of interactions on the survival of these bacteria, we harvested total cells from the entire mono and coculture plates and enumerated colony-forming units (CFU) for each bacterium using selective media. We observed a small increase in the Pa population and a decrease in the Kp population in coculture (Fig. 2C). We also harvested cells from various regions (RP, CZ, and TZ) of coculture plates of area ~38 mm^2^ at 24 h and enumerated CFU for Pa and Kp. We enumerated CFU from corresponding zones of Pa and Kp monoculture plates as respective controls. We observed that the Kp population at the radial point and clearance zone was slightly less in coculture plates than in Kp monoculture plates (Fig. 2D), consistent with them being pushed out. Interestingly, the Pa population in the clearance zone of coculture plates was more than in the Pa monoculture control plates, suggesting that some Pa cells could move into the clearance zone. In the toroid zone (TZ), where there is increased material, we detected Kp and a few Pa cells. To check if either bacterium was using bactericidal offense mechanisms against the other, we performed a live-dead assay, taking cells from different zones of the coculture plate. Although we observed 100% death in heat-killed bacteria (used as control), we observed a few dead, propidium iodide-stained cells in RP and CZ regions, suggesting negligible death in coculture plates (Fig. S3). These results indicated that Pa employs a nonbactericidal mechanism to create the clearance zone.

### Quorum sensing in *P. aeruginosa* facilitates the toroidal displacement of *K. pneumoniae*

**Fig 3:**
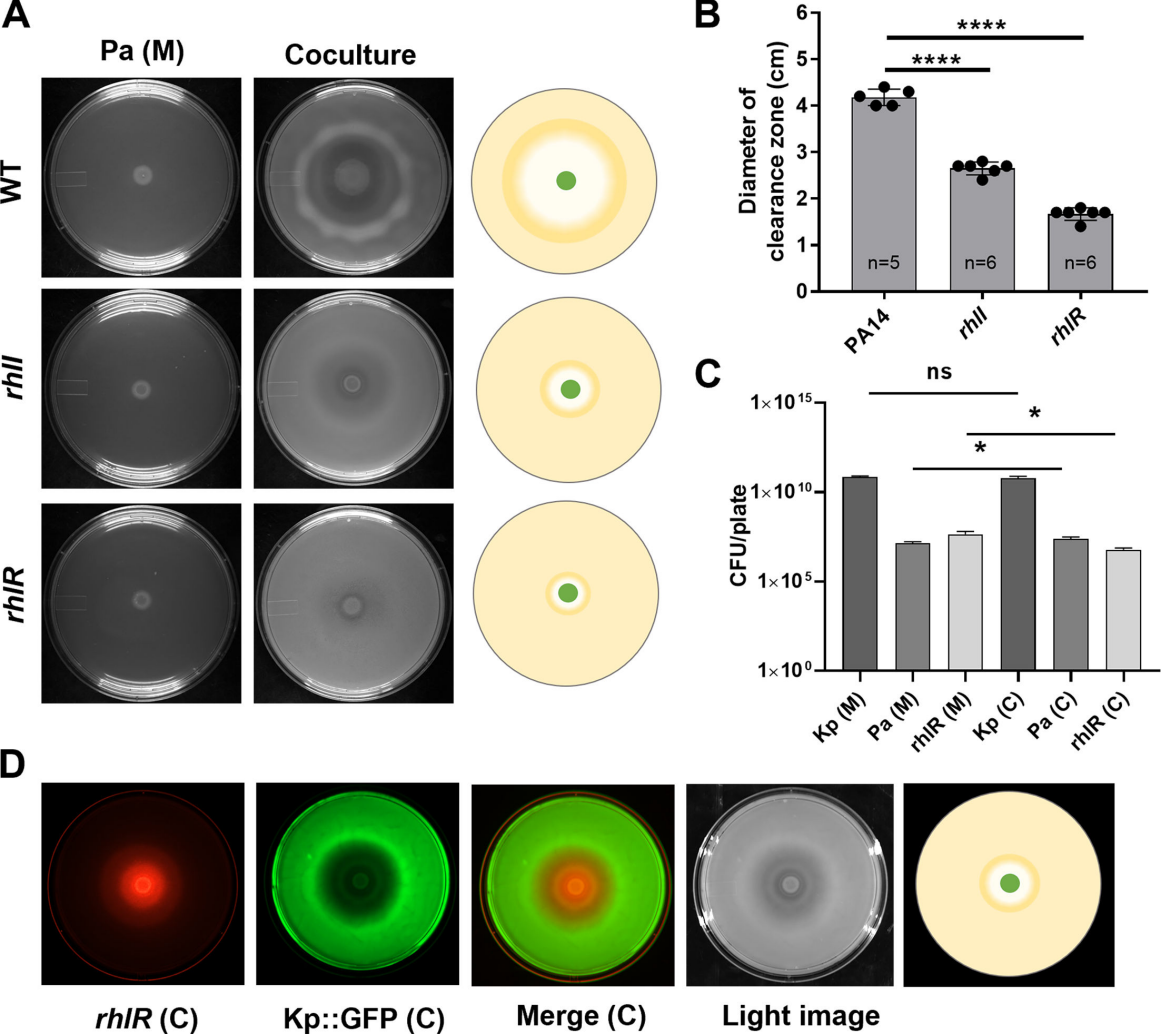
RhlR/I quorum sensing system of *P. aeruginosa* contributes to the displacement of *K. pneumoniae* in coculture. (**A**) Interaction of wild-type, *rhlR*, and *rhlI* mutants of *P. aeruginosa* with Kp on the M9 coculture assay plate. Pa (M) represents a Pa monoculture plate. (**B**) Quantification of the clearance zone created by PA14, *rhlI*, and *rhlR* mutants on M9 medium. One-way analysis of variance, followed by Dunnett’s test, was used for the analysis of significance (ns, nonsignificant; ****, *P*  ≤  0.0001). (**C**) Enumeration of the total CFU of Pa and Kp from monoculture (M) and coculture (C) plates. An unpaired *t*-test was used for the analysis of significance (ns, nonsignificant; *, *P*  ≤  0.05). (**D**) Interaction of *rhlR* mutant expressing mCherry, with Kp expressing GFP on M9 coculture plate. Error bars indicate SEM.

Since quorum sensing (QS) modulates the offense and defense strategies of *P. aeruginosa* (26), we first asked if quorum sensing is necessary for toroidal displacement. We studied the requirements of various components of hierarchical QS systems in *P. aeruginosa* (27). We found that RhlR, the transcriptional regulator of the autoinducer butanoyl homoserine lactone (C4-HSL), as well as RhlI, an enzyme for the autoinducer, was needed in Pa for the optimal displacement of the Kp population (Fig. 3A). Toroidal displacement of Kp was reduced more in the *rhlR* mutant than in the *rhlI* mutant compared to wild-type PA14 (Fig. 3B), and the difference may result from a residual activity of RhlR in the *rhlI* mutant. The phenotypes for *rhlI* and *rhlR* mutants were rescued by complementation of wild-type copies of *rhlI* and *rhlR* genes, respectively (Fig S4). Reduction in the displacement of Kp was also observed upon spotting mCherry-expressing *rhlR* mutant on GFP-expressing Kp (Fig. 3D). The reduced displacement was also reflected in CFU analysis. The total population of Kp between mono and coculture plates was similar, while there was a very small decline in the population of Pa (*rhlR*) cells in coculture plates (Fig. 3C). We also examined the requirement for the LasR/I system consisting of LasR transcriptional activator and LasI involved in the synthesis of autoinducer 3-oxo-dodecanoyl homoserine lactone (oxo-C12-HSL) (28). We observed that both LasI and LasR were dispensable for the clearance of Kp (Fig S5). Neither LasA nor neighboring LasB proteases of Pa, both under the control of the LasR/I system, were required for the clearance of Kp (Fig S5). Pseudomonas quinolone system was also dispensable, as mutations in the pqsABCDE operon did not affect the toroidal displacement of Kp (Table S2). PqsE has been shown to positively regulate RhlR in the PA01 strain (29), but it does not seem to affect RhlR-dependent displacement of Kp by the PA14 strain used in this study. Altogether, these results suggest that the RhlR/I quorum sensing system of Pa contributes to the clearance of Kp in a surface interaction regime.

### Rhamnolipids contribute to the toroidal displacement activity of *P. aeruginosa*

To understand if a specific factor(s) of *P. aeruginosa* drives the toroidal displacement of *K. pneumoniae* cells, we examined several (30) mutants of *P. aeruginosa* in spot-on-lawn interaction assays. We tested 202 transposon insertion mutants of Pa against Kp (Table S2). Surprisingly, none of the examined virulence factors influenced the process, except genes involved in the synthesis of surfactant rhamnolipid. This analysis indicated that Pa does not utilize bactericidal mechanisms for clearance of Kp, consistent with our live/dead analysis in coculture plates. Pa likely utilizes killing-independent mechanisms to push Kp cells out of the way.

**Fig 4:**
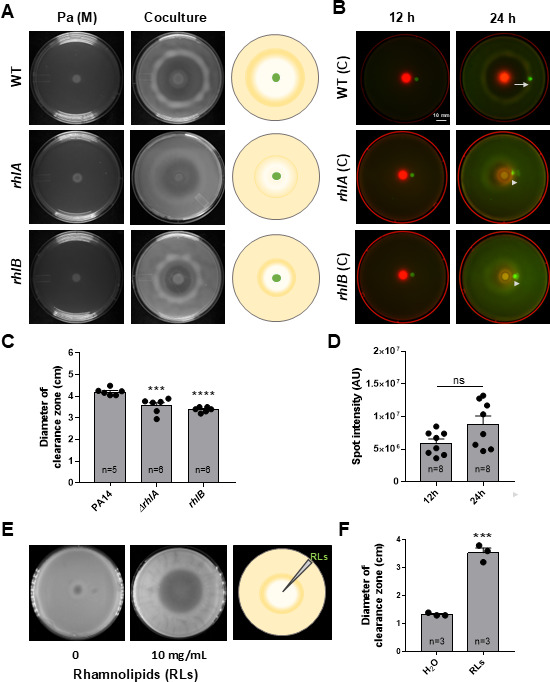
*P. aeruginosa* employs rhamnolipids for the toroidal displacement of *K. pneumoniae*. (**A**) Interaction of Pa (wild type, *rhlA*, and *rhlB)* with Kp on M9 coculture plates. Pa (M) represents a Pa monoculture plate. (**B**) Location of GFP-expressing Kp spotted 1 cm from the center of a Pa::mCherry- Kp coculture plate at 12 and 24 h of incubation. Displacement of Kp::GFP spot is indicated with an arrow in images obtained after 24 h. Scale bar, 1 cm. (**C**) Quantification of clearance zone diameter on coculture plates with PA14, *rhlA*, or *rhlB* mutant on M9 media. (**D**) Quantification of the intensity of Kp::GFP spot on the Pa-Kp coculture plate at 12 and 24 h. (**E**) Displacement of Kp cells from its lawn when water (H_2_O) or 10 mg/mL rhamnolipid is spotted in the center of the Kp lawn. (**F**) Quantification of the clearance zone diameter created by water or 10 mg/mL rhamnolipid at the center of the Kp lawn. One-way analysis of variance, followed by Dunnett’s test, was used for the analysis of significance (ns, nonsignificant, ***, *P*  ≤  0.001, ****, *P*  ≤  0.0001) in panel C. An unpaired *t*-test was used for the analysis of significance in panels D and F (ns, nonsignificant). Error bars indicate SEM.

Of the 202 factors tested, only two contributed to the displacement of Kp. We found that mutations in either *rhlA* or *rhlB* genes encoding acetyltransferase and rhamnosyltransferase I, respectively, resulted in reduced displacement of Kp (Fig. 4A and C). This indicated that rhamnolipids help Pa push Kp to the edge of the clearance zone. To distinguish between pushing versus killing, we spotted 5 µL of Kp::GFP over the lawn of unlabeled Kp (Fig. 4B) and imaged the plates at 12 and 24 h. We found that the fluorescent Kp spot was pushed out all the way to the toroid zone, but there was no decline in the fluorescence intensity of the Kp::GFP spot, suggesting there was no death of Kp (Fig. 4D). The *rhlA* and *rhlB* mutants of Pa were unable to push Kp::GFP to the same extent as wild-type Pa (Fig. 4B). The clearance zone observed on *rhlA* or *rhlB* was larger than that observed for *rhlR* (Fig. 3B), suggesting that additional factors controlled by RhlR are involved in toroidal displacement. To confirm the involvement of rhamnolipids in toroidal displacement, we performed staining with a lipophilic dye, Nile red, in mono and Pa-Kp coculture plates (31, 32). We found that Nile red-stained rhamnolipids accumulated in the toroid zone (Fig S6). To confirm the involvement of rhamnolipid, we applied exogenous rhamnolipid at the center of a lawn of Kp and found that repeated spotting of 10 mg/mL rhamnolipid resulted in the displacement of Kp cells from the center of its lawn (Movie S2; Fig. 4E and F). Altogether, these experiments provided evidence that Pa pushes Kp using rhamnolipid biosurfactant.

### Iron limitation promotes toroidal displacement action of *P. aeruginosa* against *K. pneumoniae*

**Fig 5:**
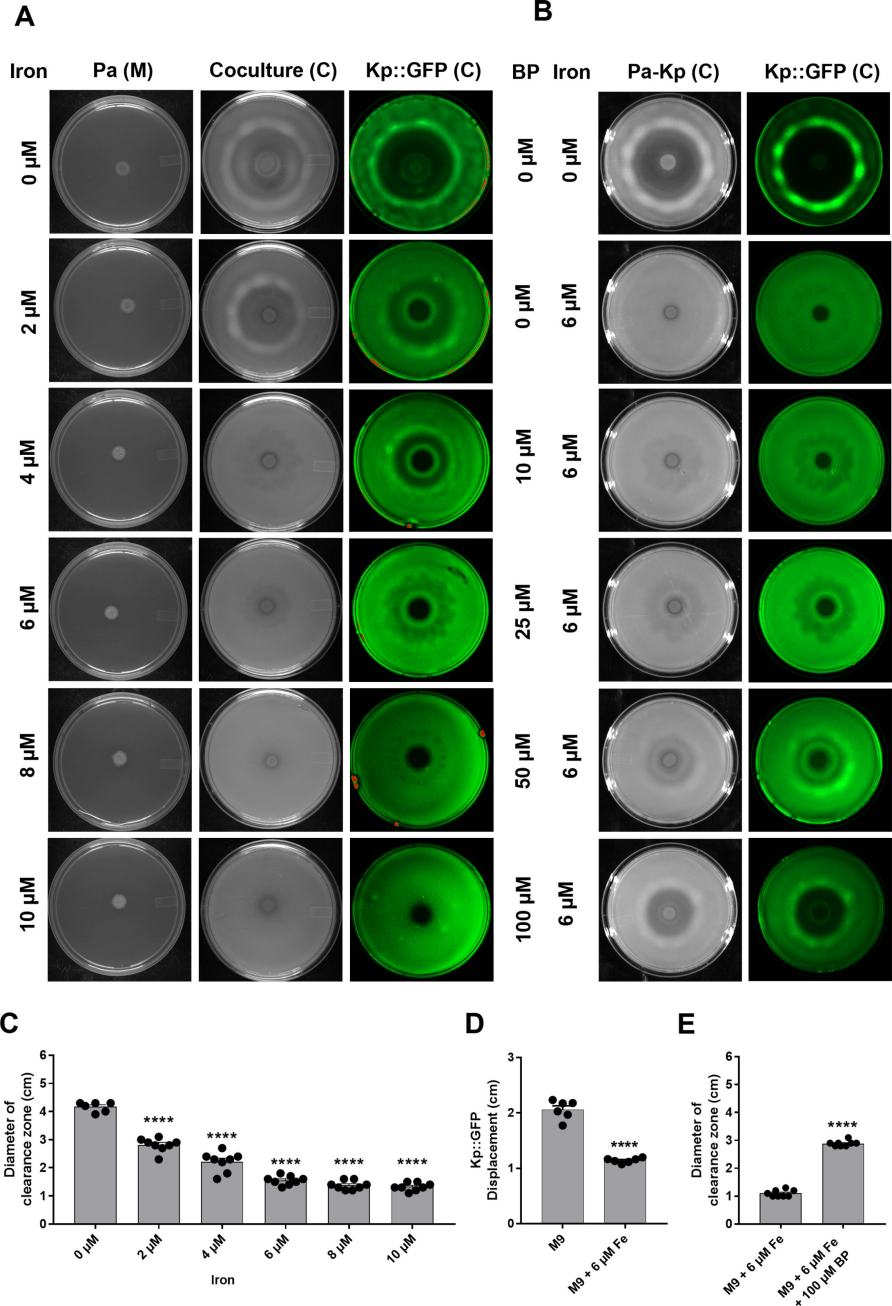
Iron limitation promotes toroidal displacement of *K. pneumoniae* by *P. aeruginosa*. (**A**) Pa-Kp coculture assays on M9 plates supplemented with 0 to 10 µM of FeSO_4_·7H_2_O. (**B**) Pa-Kp coculture assays on M9 plates supplemented with 10 µM of FeSO_4_·7H_2_O, and 0 to 125 µM of iron chelator 2,2′-bipyridyl (BP). Pa (M) represents the Pa monoculture, and Kp::GFP (**C**) represents the Pa-Kp::GFP coculture plate. (**C**) Quantification of clearance zone on M9 media with an increasing concentration of FeSO_4_. One-way analysis of variance, followed by Dunnett’s test, was used for the analysis of significance (****, *P*  ≤  0.0001). (**D**) Displacement of Kp::GFP spot on Pa-Kp coculture plates with and without 6 mM iron from images obtained after 24 h (also see Fig S8). (**E**) Quantification of clearance zone on M9 media with 6 µM FeSO_4_ with and without 100 µM bipyridyl. An unpaired *t*-test was used for the analysis of significance (****, *P*  ≤  0.0001). *n* represents biological replicates. Error bars indicate SEM.

Iron is required for respiration and growth in all living organisms, and iron limitation is a driver of virulence in *P. aeruginosa* (33–35), as well as swarming (36–38). Therefore, we asked whether iron limitation drives toroidal displacement of Kp by Pa. We examined the toroidal displacement activity on coculture plates supplemented with an increasing concentration of iron (ferrous sulfate) ranging from 2 to 10 µM in addition to un-supplemented control. We found that iron above 2 µM diminished toroidal displacement of Kp, while 10 µM iron was sufficient to completely abolish toroidal displacement (Fig. 5A). Increasing concentration of iron led to a corresponding decrease in the clearance zone (Fig. 5C). Furthermore, addition of iron led to loss of pushing of Kp::GFP spot in a Pa-Kp interaction assay (Fig S8 and Fig. 5D).

To further confirm the role of iron limitation in toroidal displacement, we added an iron chelator, 2,2′-bipyridine (BP), to iron-supplemented coculture plates. We could restore the toroidal displacement of Kp by adding 100 µM BP in M9 plates supplemented with iron (Fig. 5B and E). The analyses of toroidal displacements with additional iron and iron chelator provided strong evidence that iron limitation drives the toroidal displacement of *K. pneumoniae* by *P. aeruginosa*.

### *K. pneumoniae* enhances siderophore synthesis in *P. aeruginosa*

**Fig 6:**
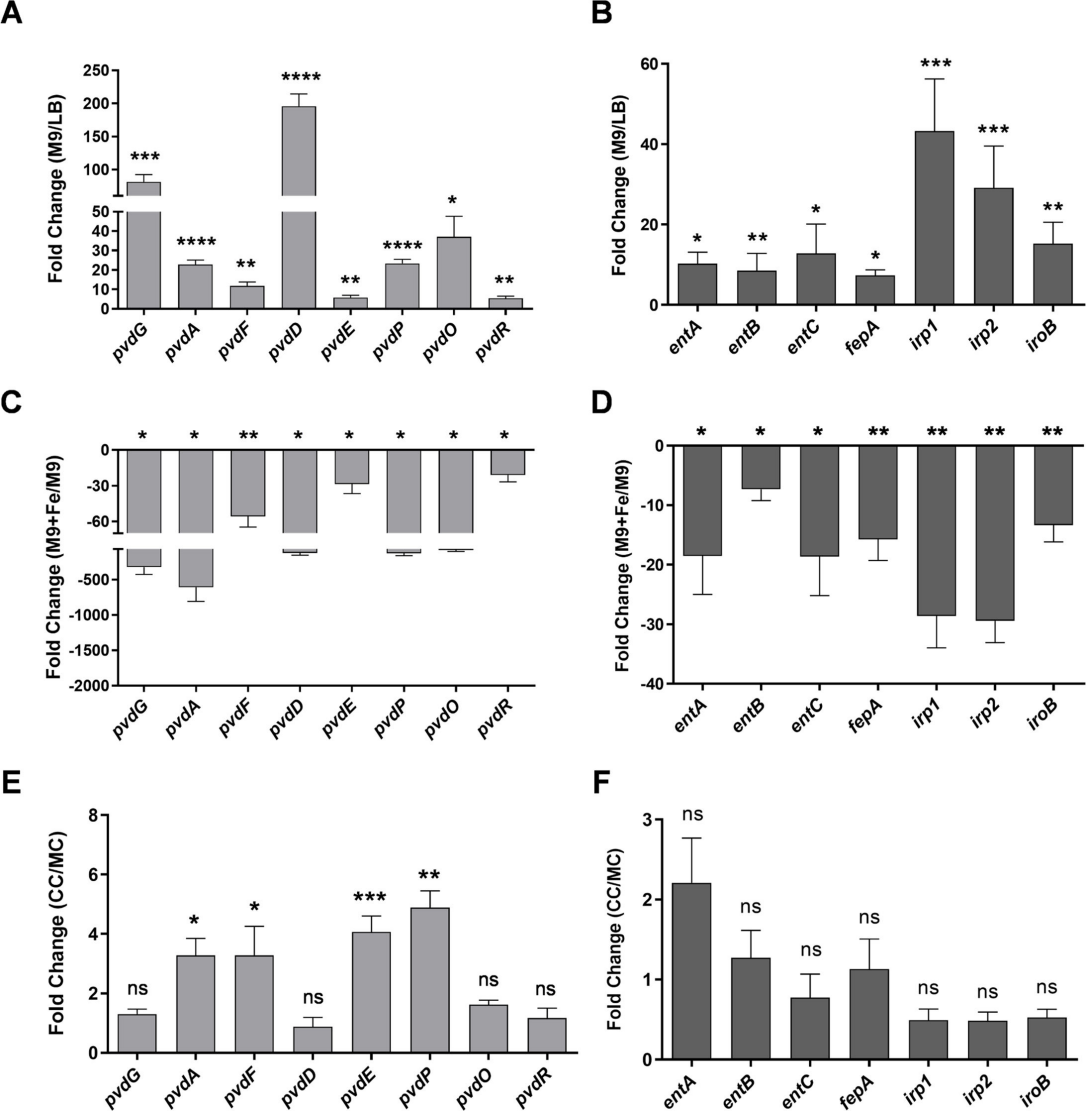
*P. aeruginosa* and *K. pneumoniae* compete for iron in M9 medium. Analysis of the expression of genes involved in siderophore synthesis and export in *P. aeruginosa* (**A**) grown in M9 medium over LB medium or (**C**) grown in iron-supplemented M9 medium over M9 medium for 12 h at 37°C. Analysis of the expression of genes involved in siderophore synthesis in *K. pneumoniae* (**B**) grown in M9 medium over LB medium or (**D**) grown in iron-supplemented M9 medium over M9 medium for 24 h at 37°C. (**E**) Analysis of expression of Pa genes involved in siderophore synthesis in coculture over Pa monoculture. (**F**) Analysis of expression of Kp genes involved in siderophore synthesis in coculture over Kp monoculture. Three biological replicates were used. An unpaired *t*-test was used for analysis of significance in panels A to F (*, *P*  ≤  0.05; **, *P*  ≤  0.01; ***, *P*  ≤  0.001, ****, *P*  ≤  0.0001, ns, nonsignificant). Error bars indicate SEM.

All living organisms, including bacteria, employ various strategies to harvest iron from the environment and their neighbors. Siderophores are small organic molecules secreted by bacteria to harvest extracellular iron (39). Pyoverdine is one of the major siderophores secreted by *Pseudomonas*. We examined the expression of various genes involved in the synthesis of pyoverdine in *P. aeruginosa* (schematic in Fig S7A). We examined the level of transcripts for *pvdG*, *pvdA*, *pvdF*, *pvdD*, *pvdE*, *pvdP*, *pvdO*, and *pvdR* in *P. aeruginosa* grown in LB or M9 medium. We found several fold upregulations in all these genes in cells grown in M9 compared to cells grown in LB medium (Fig. 6A). This indicated that the M9 medium has less iron than the LB medium, consistent with the measurement of iron in LB (Fig S9B) and inability to detect iron in minimal medium. An independent measurement of pyoverdine secreted by Pa in M9 and LB confirmed that M9 medium promotes pyoverdine production (Fig S9A). When we supplemented M9 medium with 36 µM iron, we observed suppression in the expression levels of transcripts for genes involved in siderophore synthesis in Pa (Fig. 6C).

*K. pneumoniae* is known to synthesize as many as four different siderophores, namely, enterobactin, yersiniabactin, salmochelin, and aerobactin, to establish infection (40) (schematic in Fig S6B). We examined the expression of some of the genes involved in the synthesis of enterobactin (*entA*, *entB*, *entC*, and *fepA*), salmochelin (*iroB*), and yersiniabactin (*irp1*, *irp2*), and we found that all the transcripts were upregulated in Kp grown in M9 medium over Kp grown in LB medium (Fig. 6B). This upregulation was suppressed when Kp cells were grown in M9 medium supplemented with 36 µM of iron (Fig. 6D). These experiments suggested that both Pa and Kp respond to iron-limiting conditions in the M9 medium by upregulating genes involved in the production of various siderophores.

To understand the importance of iron in driving interbacterial interactions, we measured total iron available in M9 and LB media using an iron estimation kit. While iron was undetectable in the M9 medium, there was up to 2 µM of free iron in the LB medium. To get an insight into the ability of Pa and Kp to utilize iron, we grew Kp and Pa individually in the LB medium or in M9 supplemented with 100 M iron and measured iron in the spent media after 6, 12, and 16 h of growth. As shown in Fig S9B, both bacteria depleted iron in a time-dependent manner. To reduce the competition for iron, we studied the *ybtU* mutant of Kp (lacking yersiniabactin, one of the four siderophores). We confirmed that the *ybtU* mutant had a reduced ability for iron uptake compared to the parental strain KPPR1 (Fig S10). We also quantified the displacement of Kp::GFP by Pa and found that displacement was lower on the *ybtU* mutant of Kp (Fig S10). As expected, the clearance zone for *ybtU* was smaller than the clearance of parental KPPR1 in coculture plates (Fig S10).

Altogether, our experiments indicate that iron competition alters the behavior of *P. aeruginosa* in a manner that allows it to push *K. pneumoniae* out of its way.

### Rhamnolipid production induced by iron limitation regulates *P. aeruginosa* behavior on solid surfaces

**Fig 7:**
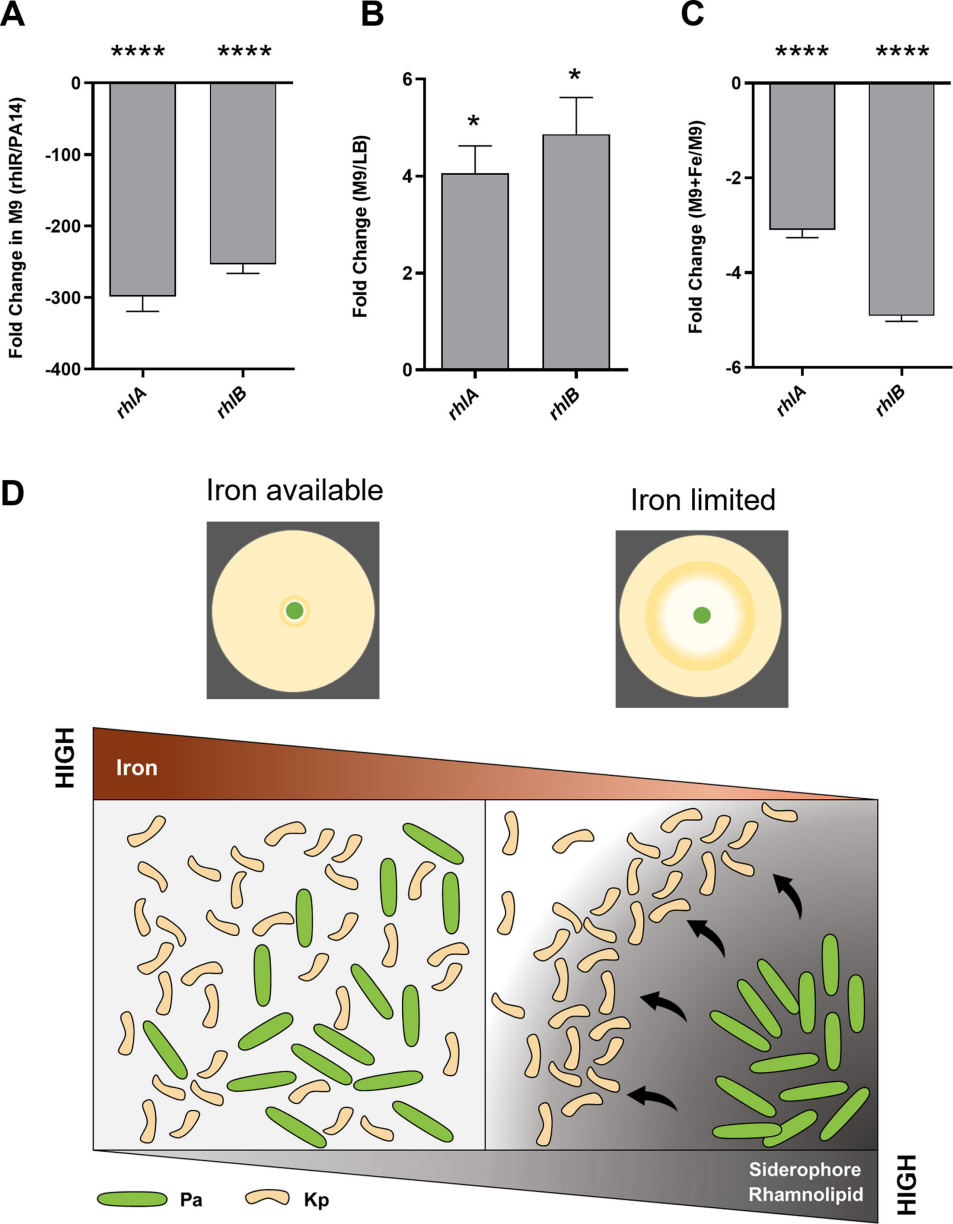
Rhamnolipid production induced by iron limitation regulates *P. aeruginosa* behavior on surfaces. (**A**) Analysis of expressions of *rhlA and rhlB* genes of *P. aeruginosa* after 24 h of growth in rhlR mutant and PA14 in M9 medium (**B**) in PA14 grown in M9 over LB medium, and (**C**) in PA14 grown in iron-supplemented M9 medium over M9 medium alone. An unpaired *t*-test was used for the analysis of significance (*, *P*  ≤  0.05; ****, *P*  ≤  0.0001). Four biological replicates were used. Error bars indicate SEM. (**D**) Model for the interaction between *P. aeruginosa* and *K. pneumoniae.* These bacteria can co-exist in nutrient-replete conditions. Under iron-limiting conditions, siderophore synthesis is induced in both bacteria. Iron limitation can also turn on the synthesis of biosurfactants, rhamnolipids, in *P. aeruginosa,* allowing it to push away *K. pneumoniae***.**

Our study suggested that iron limitation is the driving force for toroidal displacement of Kp by Pa. We also observed that Pa employs a rhamnolipid-dependent mechanism to push Kp to the toroid zone. The connection between iron limitation and increased rhamnolipid levels has been well established previously (36, 41–45). We asked if iron limitation also drives rhamnolipid synthesis during interaction between Pa and Kp. We analyzed the expression of transcripts for rhamnosyl transferases, *rhlA* and *rhlB*, in Pa cells grown in LB and M9. We confirmed that the *rhlR* mutant is defective in producing rhamnolipid in M9 media. As shown in Fig. 7A, *the rhlR* mutant had reduced expression of transcripts for *rhlA* and *rhlB*, as expected. We then established the effect of media and iron supplementation on rhamnolipid levels. By comparing Pa cells grown in M9 and LB media, we found that expressions of *rhlA* and *rhlB* were upregulated in M9-grown Pa (Fig. 7B). Furthermore, we observed that iron supplementation in the M9 medium completely suppressed the upregulation of *rhlA* and *rhlB* (Fig. 7C). Altogether, our study provided strong evidence that iron limitation allows *P. aeruginosa* to increase rhamnolipid levels to push *K. pneumoniae* out of its way to limit the competition for iron. A model for the interaction of *P. aeruginosa* and *K. pneumoniae* under iron-limiting conditions is shown in Fig. 7D.

## DISCUSSION

Studies on the interactions of *P. aeruginosa* with its neighbors have shown that it employs a range of offense and defense mechanisms, including the use of soluble toxic molecules, such as phenazines, hydrogen cyanide, proteases, phospholipases against gram-positive bacteria, and pathogenic yeast (1, 46–51). In this study, we have shown that under specific conditions, *P. aeruginosa* does not utilize bactericidal mechanisms. Rather, it adopts a unique pushing mechanism to displace *K. pneumoniae*, a fierce scavenger of iron. Our study shows that *P. aeruginosa* can co-exist with *K. pneumoniae* in rich media, but under the condition of iron limitation, it pushes *K. pneumoniae* out using rhamnolipid biosurfactant, providing evidence for context-dependent behavior modulation among neighbors.

Our study found no evidence that *P. aeruginosa* deploys bactericidal mechanisms against *K. pneumoniae*. Why should this be so? One explanation might be that *K. pneumoniae* is harmless to *P. aeruginosa.* There is no evidence that Kp kills Pa in the spot-on-lawn interaction assay. It is then likely that *P. aeruginosa* does not perceive *K. pneumoniae* as a foe.

Why does iron limitation drive toroidal displacement response from *P. aeruginosa*? While most organisms have ways to harvest iron from their surroundings, *P. aeruginosa* and *K. pneumoniae* excel at iron scavenging. In response to low Fe^2+^, Pa produces a high-affinity siderophore, called pyoverdine, allowing it to scavenge Fe^3+^ from the environment and promote its own growth (34, 52). Most *Klebsiella* species also have an impressive repertoire of at least three siderophores: enterobactin, salmochelin, and yersiniabactin (53). These two bacteria rapidly utilize iron in their surroundings (Fig S9), setting off competition for iron in minimal media. Depletion of iron not only drives siderophore synthesis in *P. aeruginosa* (Fig. 6A and C) but also induces the synthesis of rhamnolipids (Fig. 7A and B). Thus, the biosurfactant circuit has evolved to be responsive to iron availability, allowing *P. aeruginosa* to use the surfactant for driving competitors for iron away. Rhamnolipids are used for biofilm dispersal (54), antimicrobial activity (55), attenuation of host immunity (56), alteration of physical environments (57), and swarming (i) during nitrogen limitation, (ii) in low nutrient media, and (iii) during iron limitation (24, 36, 58). Our study shows that the biosurfactant activation circuit, earlier shown for swarming, is also activated during resource competition. Thus, rhamnolipid produced during iron limitation has at least two end goals: (i) the previously described role in swarming and (ii) a novel role in the pushing of competitors, such as *K. pneumoniae*, away under conditions examined in this study.

While *P. aeruginosa* and *K. pneumoniae* are two of the fierce scavengers of iron, other microbes, such as *S. aureus* and *E. coli*, can also take up iron (59, 60). We found relatively less depletion of iron by *S. aureus* and *E. coli* (Fig S9C). The lower iron-scavenging capacity of these microbes probably explains the lack of toroidal displacement in their interaction with *P. aeruginosa*. If *P. aeruginosa* does not experience iron limitation, there will not be enough surfactants to cause toroidal displacement. This is consistent with our experiments with the siderophore-deficient *K. pneumoniae* strain, *ybtU.* A slight reduction in the iron-scavenging capacity of this mutant had a measurable impact on toroidal displacement. This strain of *K. pneumoniae* utilized iron at a slower pace and consequently experienced delayed toroidal displacement response from *P. aeruginosa*.

Microbial biosurfactants are expensive to make but facilitate major processes, such as (i) the dispersal of bacteria from biofilms allowing them to colonize new niches and (ii) reduction of surface tension for swarming motility. They help in the modulation of the environment as shown recently (61). Our study suggests yet another role for the surfactant of *P. aeruginosa*. The rhamnolipids aid in the competitive fitness of *P. aeruginosa* by reducing competition for nutrients, such as iron. *P. aeruginosa* likely utilizes its surfactant to reduce adhesion between *K. pneumoniae* cells with the agar substratum. It is possible that *P. aeruginosa* can utilize surfactants to perform toroidal displacement in response to competition for other nutrients besides iron. A careful analysis of *P. aeruginosa* behaviors under various physiologically relevant, nutrient-limiting conditions will elucidate environmental features that impinge on biosurfactant production in bacteria.

## MATERIALS AND METHODS

### Microbes and growth conditions

*P. aeruginosa* PA14 (WT) and the isogenic transposon-insertion mutant strains (62) were obtained from Prof. Frederick Ausubel. Additional bacterial strains used in this study are listed in Table S1 and S2. Unless otherwise mentioned, PA14 was routinely cultured in LB broth at 37°C, while the mutants were cultured in LB broth with 50 µg mL^−1^ gentamicin. Similarly, *E. coli* strains used for cloning experiments were routinely cultured in LB broth at 37°C with appropriate antibiotics. The *K. pneumoniae* KPPR1 strain and the transposon insertion mutant *ybtU* were obtained from Prof. Harry Mobley (63). KPPR1 was cultured in LB broth at 37°C, while the transposon-insertion mutants were cultured in LB broth with 50 µg mL ^−1^ kanamycin. ICC8001 and ICC8001::GFP (Kp::GFP) were derived from KPPR1 as described (64). They were cultured in LB with 50 µg mL^−1^ carbenicillin at 37°C. *S. aureus*, *E. coli*, or *P. mirabilis* were cultured in LB at 37°C. Additional media used in this study include BHI (HiMedia) and M9 (8.6 mM NaCl, 20 mM NH_4_Cl, 1 mM CaCl_2_, 1 mM MgSO_4_, 22 mM KH_2_PO_4_, 12 mM Na_2_HPO_4_, 0.2% glucose, and 0.5% casamino acids). M8 is a modification of the M9 medium (excluding NH_4_Cl and CaCl_2_ salts) (23). Fe_2_SO_4_·7H_2_O was used as the source of iron in all the experiments involving iron. Iron chelator, 2,2′-bipyridine (Sigma-Aldrich, catalog number 8.20158) was used at indicated concentrations.

### Spot-on-lawn interaction assay

Kp lawn was made using 1:100 dilution of a 0.5 OD_600_ culture of Kp prepared from an overnight culture. Accordingly, 500 µL of the diluted culture was spread on LB, BHI, M9, or M8 medium plates solidified with 1% Bacto agar (BD), and excess liquid was removed with a pipette. The lawn was allowed to dry for 40 min at room temperature. Next, 5 µL of 2.0 OD_600_ culture of Pa was spotted at the center of the Kp lawn (coculture) or a plain plate (monoculture). Plates were dried for 20 more minutes and then incubated at 37°C for interaction to take place. Both mono and coculture plates were imaged in the Vilber E-box after 24 h of incubation. Images involving fluorescence were captured in the Bio-Rad ChemiDoc MP system. On a given day, at least three plates were used for each monoculture and coculture. Two to four biological replicates were performed each on a different day. All Kp strains (KPPR1, ICC8001, and ICC8001:GFP) were displaced to the same extent by wild-type PA14 (Fig S11). This includes names of Pa mutants used in the interaction assay with Kp along with images of mono and interaction plates.

To examine the effect of rhamnolipids on the displacement of *K. pneumoniae* cells, a rhamnolipid mixture (Sigma-Aldrich, catalog number R90) (10 mg/mL in water) was applied to the center of a 16 h-old lawn of Kp in 10 µL volumes for three times. As a control, water was applied to a lawn of Kp.

### Colony-forming unit assay

CFU were estimated in coculture or monoculture plates by washing off the plate with phosphate-buffered saline (PBS). Serial dilutions were made and plated on cetrimide agar (HiMedia, catalog number M0124) and HiCrome *Klebsiella* selective agar base (HiMedia, catalog number M1573) to enumerate Pa and Kp, respectively. To estimate the numbers of Pa and Kp in different zones (radial point, clearance zone, toroid zone) (Fig. 2B) in coculture, a 38 mm^2^ plug of agar was captured using the back of a 1 mL tip. The bacteria from the plug were diluted using PBS and plated on selective agar to enumerate colonies of Pa and Kp.

### Live-dead staining assay

Wild-type Pa and Kp lacking fluorescence markers were used for these assays. Bacterial cells were collected from different zones of mono and coculture plates after 24 h of incubation. A Live/Dead Double Staining Kit (Merck Life Sciences, catalog number 04511) was used to stain cells, followed by microscopy. Heat-killed Kp cells are used as a control. After staining, cells were imaged using a Stedycon optical setup (Abberior Instruments GmbH) integrated with a Leica SP 5 microscope (Leica Microsystems GmbH, Manheim, Germany).

### Nile red staining

To visualize rhamnolipids, M9 agar plates containing Nile red are prepared by adding 10 µg mL^−1^ Nile red (MP Biomedicals) (31, 32). Images of plates were captured in the Bio-Rad ChemiDoc MP System using a rhodamine filter.

### Pyoverdine estimation

A 1.0 OD_600_ Pa or Δ*pvdE* culture was inoculated in M9 and LB broth at 1% inoculum. After growth for 24 h at 37°C with shaking (180 rpm), cell-free supernatants were harvested. Pyoverdine fluorescence was measured in the supernatants using the TECAN Infinite M200PRO microplate reader (65) (Ex/Em; 365/460 nm).

### Iron estimation

For iron estimation, 1% inoculum of 1.0 OD_600_ Pa and Kp culture was inoculated in LB broth. Total iron was measured in the spent media after 6, 12, and 16 h of bacterium growth for 24 h at 37°C with shaking (180 rpm) using an iron assay kit (Sigma-Aldrich, catalog number MAK025). A TECAN infinite M200PRO microplate reader was used for measuring the colorimetric readings. To determine the relative iron-scavenging capacity of bacteria, we allowed bacteria (10^4^ cells/mL) to grow in M9 medium supplemented with 100 µM FeSO_4_ for 30 min at 37°C. The total iron in the cell-free spent medium was measured for *P. aeruginosa* PA14, *K. pneumoniae* KPPR1, *E. coli*, and *S. aureus*. The estimation was done with four replicates for each bacterium, and at least two biological replicates were performed, each on a different day. Iron could not be detected in unsupplemented M9 medium.

### RNA preparation

Total RNA was extracted from a final volume of 2 mL of harvested cells from an overnight bacterial culture grown in M9 and M9 supplemented with Fe (normalized to OD_600_ of 1) by the hot-phenol method as described (66). Briefly, cells chilled on the ice were centrifuged at 5,000 rpm for 5 min. The pellet was resuspended in the lysis buffer (20 mM Tris-HCl [pH 7.5], 20 mM NaCl, 5 mM Na_2_EDTA, 5 mM VRC, SDS (1% [wt/vol]) and 2-mercaptoethanol [0.7% [vol/vol]) to which equal volume of hot phenol (pH 4.3, 65°C) was added and vortexed, followed by incubation at 65°C for 6 min. Samples were centrifuged at 12,000 rpm for 15 min to recover the aqueous layer, which was further extracted using an equal volume of phenol:chloroform mixture (12,000 rpm*,* 10 min), followed by an equal volume of chloroform (12,000 rpm*,* 7 min). The RNA was precipitated with ethanol and resuspended in 0.5 mL of molecular-grade water. Subsequently, RNA purity was analyzed spectrophotometrically (NanoDrop 1000) and subjected to DNase I treatment to remove genomic DNA contamination. Next, 10 µg of RNA was used for DNase treatment at 37°C for 30 min, followed by RNA precipitation by the phenol-chloroform method. Finally, the RNA was resuspended in nuclease-free water and quantified.

### qRT-PCR analysis

RNA was treated with DNase I and quantified on a NanoDrop spectrophotometer (ND-1000). Next, 1 µg of RNA was used for cDNA preparation using iScript cDNA Synthesis Kit (Bio-Rad, catalog number 1708891). The iTaq Universal SYBR Green Supermix (Bio-Rad, catalog number 1725120) was used for qRT-PCR on the Quant Studio 3 Real-Time PCR System (Applied Biosystems). We used at least three biological replicates for each sample and two technical replicates for each biological replicate. Fold change for each transcript was calculated from the *C*_*⊤*_ values normalized to the housekeeping gene *rpoD* following the 2^−ΔΔCT^ method (67). Primers used for qRT-PCR are described in Table S1.

### Construction of deletion and complementation strains of *P. aeruginosa* PA14

A markerless deletion strain of *pvdE* was generated by the two-step allelic exchange strategy (68). Clones were screened by deletion-specific PCR and confirmed by sequencing. See Table S1 for primers used for the construction of pEXG2-Δ*pvdE.* The complementation of *rhlA*, *rhlI*, and *rhlR* (denoted as *rhlA+*, *rhlI+*, and *rhlR+*) was carried out as previously described (69). Entire gene, including the promoter region, was PCR-amplified from PA14 genomic DNA using primer pairs (Table S1) and cloned into the mini-CTX-1 (70) vector carrying tetracycline resistance cassette. Individual constructs were transformed into *E. coli* SM10. The complementation construct was moved from *E. coli* SM10 donor to the Δ*rhlA*, *rhlI*::Tn, and *rhlR*::Tn strains of *P. aeruginosa* using a conjugative transfer approach. Transformants were chosen on LB plates containing 50 µg/mL tetracycline. Complementation was confirmed by genotyping.

### Statistics

All experiments were performed in three or more biological replicates (*N*), unless otherwise indicated. An unpaired *t*-test was used for comparison between the control and test groups for qRT-PCR. For comparisons of clearance zones, one-way analysis of variance with Tukey’s or Dunnett’s *post-hoc* test was used as indicated in respective figure legends.

## Data Availability

The Δ*pvdE*, Δ*rhlA*, *rhlI*+, *rhlR*+, and *rhlA*+ strains used in this study are available upon request. All data are present in the paper, supplemental information, and source files for individual figures.

## SUPPLEMENTAL MATERIAL

Legends mbio.01149-25-s0001.docx Legends for Movies S1 and S2, Tables S1 and S2, and Figures S1 to S11.

Movie S1 mbio.01149-25-s0002.avi *P. aeruginosa* monoculture spot, *K. pneumoniae* monoculture lawn, and *P. aeruginosa* spot on a *K. pneumoniae* lawn.

Movie S2 mbio.01149-25-s0003.avi Displacement of *K. pneumoniae* cells by rhamnolipid.

Table S1 mbio.01149-25-s0004.xlsx Reagents, bacterial strains, and primers used in the study.

Table S2 mbio.01149-25-s0005.pdf Spot-on-Kp lawn assay for 202 transposon insertion mutants of *P. aeruginosa* PA14.

Supplemental Figures mbio.01149-25-s0006.pdf Figure S1 to S11.

ASM does not own the copyrights to Supplemental Material that may be linked to, or accessed through, an article. The authors have granted ASM a non-exclusive, world-wide license to publish the Supplemental Material files. Please contact the corresponding author directly for reuse.
