# Supplementary material for: Toroidal displacement of Klebsiella pneumoniae by Pseudomonas aeruginosa is a unique mechanism to avoid competition for iron
Source: mBio. 2025 Jun 11;16(7):e01149-25. doi: 10.1128/mbio.01149-25 (PMC12239573; doi:10.1128/mbio.01149-25)
Supplement: Legends — for Movies S1 and S2, Tables S1 and S2, and Figures S1 to S11. [file mbio.01149-25-s0001.docx]

**Toroidal displacement of *Klebsiella pneumoniae* by *Pseudomonas aeruginosa* is a unique mechanism to avoid competition for iron**

Diana Pradhan, Ajay Tanwar, Joshua Wong, Srividhya Parthasarathi, Gad Frankel, Varsha Singh

Lead author: [Vsingh001@dundee.ac.uk](mailto:Vsingh001@dundee.ac.uk)

Legends for supplementary material

Movie S1

Movie S2

Tables, S1-S2

Figures, S1-S11

**Movie S1**: Plates containing a spot of *P. aeruginosa* (Pa), a lawn of *K. pneumoniae* (Kp) and Pa spot-on-Kp lawn co culture assay. Movies for all three plates were performed for 23 hours of incubation at 37 °C. A time counter is displayed in the top left corner.

**Movie S2**: A plate containing a 16-hour lawn of *K. pneumoniae* (Kp) was spotted with10mg/ml of rhamnolipid three times at 0, 10 and 15 minutes at 37 °C. A time counter is displayed in the top left corner.

**Table S1**: Reagents, bacterial strains and primers used in the study

**Table S2**: Spot on Kp lawn assay for 202 transposon insertion mutants of *P. aeruginosa* PA14.

**Figure S1: Toroidal zone formation is due to the activity of *P. aeruginosa*.** (A) Kp spot over Pa lawn in coculture plate. Pa lawn and Kp spot on plain agar plate is used as monoculture (M) control.  (B) Three different bacteria i.e., *S. aureus*, *E. coli*, and *P. mirabilis* were spotted on Kp lawn. All three bacteria were spotted individually on plain agar plate for monoculture control. Images were acquired after 24 hours of incubation at 37 °C.

**Figure S2: Toroidal displacement by *P. aeruginosa* is a specific response *P. aeruginosa* against *K. pneumoniae*.** PA14 was spotted on the lawns of *K. pneumoniae*, *E. coli, Proteus mirabilis, Salmonella Typhimurium,* and *Serratia marcescens* on M9 media solidified with 1.5% agar. Plates were imaged after 24 hours of incubation at 37 °C.

**Figure S3: Live-dead assay shows no sign of killing of *K. pneumoniae* by *P. aeruginosa*.** Live/dead staining was performed on bacteria population taken from different zones. Heat killed (Positive control), spot control (Pa population), lawn control (Kp lawn population), radial point (bacteria from centre of coculture plate), Clearance zone (bacteria taken from clearance zone), toroid zone (bacteria taken from toroid zone).

**Figure S4: Complementation of wild type alleles in the QS mutants restored toroidal displacement of *K. pneumoniae*.** Complementation in *ΔrhlA* (A), *rhlI* (B) and *rhlR* (C) rescued the toroidal displacement phenotype. Plate images were acquired after 24 hours of incubation at 37 °C. n represents biological replicates. ****P ≤ 0.0001 as determined by Student’s *t*-test. Error bars indicates SEM.

**Figure S5: LasR/I quorum sensing system and its effector toxins are not required for *K. pneumonie* displacement.** *lasR/I* quorum sensing mutants *ΔlasA, lasI, lasB, lasA* were spotted on Kp lawn and incubated at 37 °C for 24 hours. All three bacteria were individually spotted on plain agar plate for monoculture plate control.

**Figure S6: Rhamnolipid accumulation in the toroid zone on Pa-Kp spot-on-lawn interaction plate.** Rhamnolipids are stained using a lipophilic dye, Nile red. White arrow indicates Pa cells spotted in the center and blue arrow indicates toroidal zone. Quorum sensing mutant, *rhlR*, defective in rhamnolipid synthesis is used as control.

**Figure S7: Schematic representation of siderophore synthesis pathways.**

(A) An overview of siderophore synthesis pathway in *P. aeruginosa*.

I. Synthesis of Pyoverdine precursor (Ferribactin), II. Import of ferribactin into periplasm,  III. Pyoverdine maturation, IV. Pyoverdine secretion, V. Iron chelation, VI. Uptake of PvdI-Fe^+3^, VII. Dissociation of pvdI and iron (Iron reduction), VIII. Recycle, IX. Fe^+2^ import into cytoplasm. *pvdS* is a sigma factor that regulates pyoverdine biosynthesis genes.

(B) *K. pneumoniae* siderophores synthesis pathway. All three siderophore produced by KPPR1 strain are synthesized from Chorismate (Shikimate pathway).

**Figure S8:** **Iron supplementation prevents toroidal displacement of Kp by Pa.** Location of GFP expressing Kp, spotted 1 cm from the center of a Pa::mCherry- Kp coculture plate, at 12 hours and 24 hours of incubation in plates with 6 μm iron.

**Figure S8:** **Iron supplementation prevents toroidal displacement of Kp by Pa.** Location of GFP expressing Kp, spotted 1 cm from the center of a Pa::mCherry- Kp coculture plate, at 12 hours and 24 hours of incubation in plates with 6 μm iron.

**Figure S9: Pyoverdine and iron measurement from bacterial cultures.** (A) Fluorescence for Pyoverdine was measured in cell-free supernatant at 365/460 nm after growing PA14 and *ΔpvdE in* M9 and LB broth at 37°C for 24 hours. (B) Total iron was estimated in the supernatant of *P. aeruginosa* (Pa) and *K. pneumoniae* (Kp) grown in LB at 8h, 12h and 16h post inoculation. (C) Iron was estimated in cell free spent media of *P. aeruginosa, K. pneumoniae, E. coli,* and *S. aureus* in M9 supplemented with 3 µg/ml of iron externally. Data represents 3 to 4 biological replicates. An unpaired student’s *t*-test was used for analysis of significance for A and C. One-way ANOVA followed by Dunnett’s multiple comparison test was used for B. Error bar indicates SEM. (*, P ≤ 0.01; **, P ≤ 0.01; ***, P ≤ 0.001, ****, P ≤ 0.001).

**Figure S10.** (A) Interaction of Pa with Kp (wild type and *ybtU*) on M9 coculture plates. Location of GFP expressing Kp, spotted 1 cm from the center of a Pa-Kp or Pa-*ybtU* M9 coculture plate, at 12 hours and 24 hours of incubation at 37°C. Scale bar, 1 cm. (B) Quantification of total iron in the cell-free supernatant of *K. pneumoniae* KPPR1 (Kp) and *ybtU* mutant grown in LB broth supplemented with iron for 8h. (C) Displacement of a spot of GFP expressing Kp on Pa-Kp, Pa-*ybtU* coculture plates after 24 hours. (D) Quantification of clearance zone on Pa-Kp and Pa-*ybtU* coculture plates after 24 hours. An unpaired *t*-test was used for analysis of significance in A, B, C, D, F, G and H (*, P ≤ 0.05; **, P ≤ 0.01; ***, P ≤ 0.001).

**Figure S11:** (A) Toroidal displacement of *P. aeruginosa* against KPPR1, ICC8001 and ICC8001::GFP. (B) Quantifications of clearance zone for KPPR1, ICC8001 and ICC8001::GFP. n represents biological replicates. Significance was determined by one-way ANOVA, followed by Tukey’s post hoc test (ns, non-significant). Error bars indicate SEM.
