# Supplementary material for: Toroidal displacement of Klebsiella pneumoniae by Pseudomonas aeruginosa is a unique mechanism to avoid competition for iron
Source: mBio. 2025 Jun 11;16(7):e01149-25. doi: 10.1128/mbio.01149-25 (PMC12239573; doi:10.1128/mbio.01149-25)
Supplement: Table S2 — Spot-on-Kp lawn assay for 202 transposon insertion mutants of P. aeruginosa PA14. [file mbio.01149-25-s0005.pdf]

Sr. No.	Gene	PA14 locus Tag	Description	Pa Mono-culture	Pa-Kp Coculture
1	*algP*	PA14_69370	Alginate biosynthesis	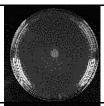	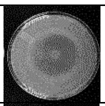
2	*alg44*	PA14_18550	Alginate biosynthesis	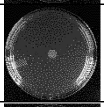	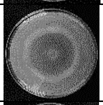
3	*algB*	PA14_72380	Alginate biosynthesis	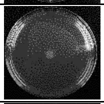	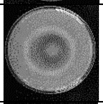
4	*algX*	PA14_18480	Alginate biosynthesis	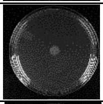	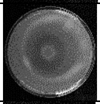
5	*algW*	PA14_57760	Alginate biosynthesis	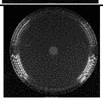	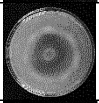
6	*algZ*	PA14_69480	Alginate biosynthesis	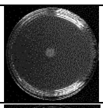	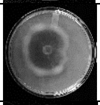
7	*algI*	PA14_18450	Alginate biosynthesis	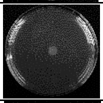	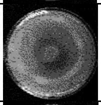
8	*algJ*	PA14_18430	Alginate biosynthesis	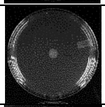	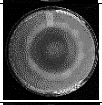
9	*algL*	PA14_18470	Alginate biosynthesis	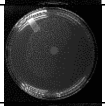	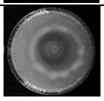
10	*algF*	PA14_18410	Alginate biosynthesis	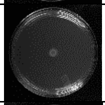	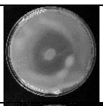
11	*algA*	PA14_18380	Alginate biosynthesis	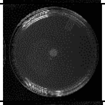	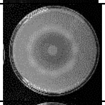
12	*algU*	PA14_54430	Alginate biosynthesis	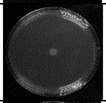	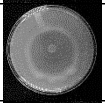
13	*algQ*	PA14_69390	Alginate biosynthesis	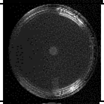	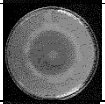
14	*algG*	PA14_18500	Alginate biosynthesis	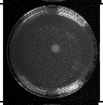	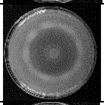
15	*algR*	PA14_69470	Alginate biosynthesis	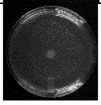	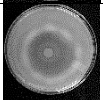
16	*rhlB*	PA14_19110	Rhamnolipid biosynthesis	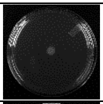	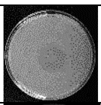
17	*rhlA*	PA14_19100	Rhamnolipid biosynthesis	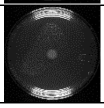	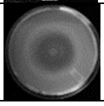

18	*fleN*	PA14_45640	Flagellar biosynthesis	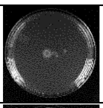	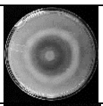
19	*fliL*	PA14_45810	Flagellar biosynthesis	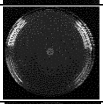	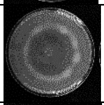
20	*fliH*	PA14_50110	Flagellar biosynthesis	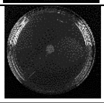	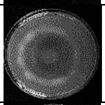
21	*flgC*	PA14_50470	Flagellar biosynthesis	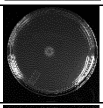	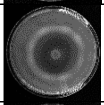
22	*flhB*	PA14_45720	Flagellar biosynthesis	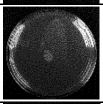	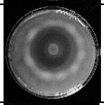
23	*flhF*	PA14_45660	Flagellar biosynthesis	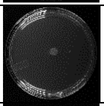	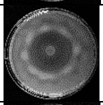
24	*fleR*	PA14_50180	Flagellar biosynthesis	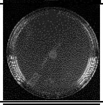	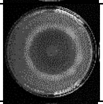
25	*flgD*	PA14_50460	Flagellar biosynthesis	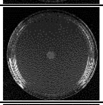	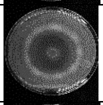
26	*flhA*	PA14_45680	Flagellar biosynthesis	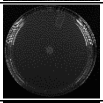	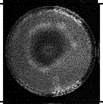
27	*fliP*	PA14_45770	Flagellar biosynthesis	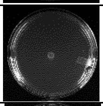	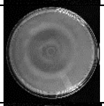
28	*fliG*	PA14_50130	Flagellar biosynthesis	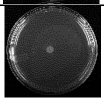	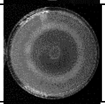
29	*fliD*	PA14_50270	Flagellar biosynthesis	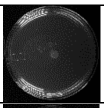	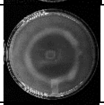
30	*fliN*	PA14_45790	Flagellar biosynthesis	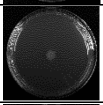	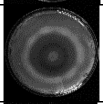
31	*flgA*	PA14_20740	Flagellar biosynthesis	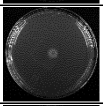	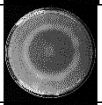
32	*fliI*	PA14_50100	Flagellar biosynthesis	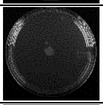	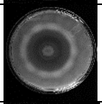
33	*fliC*	PA14_50290	Flagellar biosynthesis	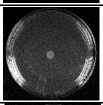	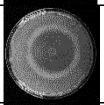
34	*fliM*	PA14_45800	Flagellar biosynthesis	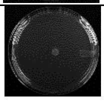	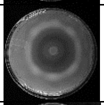
35	*fliF*	PA14_50140	Flagellar biosynthesis	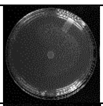	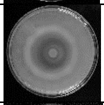
36	*fleQ*	PA14_50220	Flagellar biosynthesis	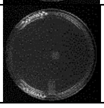	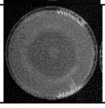

37	*fleS*	PA14_50200	Flagellar biosynthesis	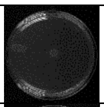	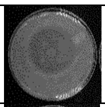
38	*flgB*	PA14_50480	Flagellar biosynthesis	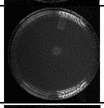	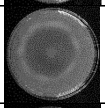
39	*flgL*	PA14_50340	Flagellar biosynthesis	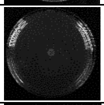	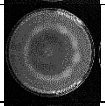
40	*fliQ*	PA14_45760	Flagellar biosynthesis	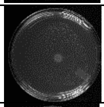	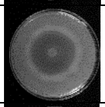
41	*flgH*	PA14_50420	Flagellar biosynthesis	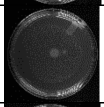	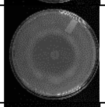
42	*flgI*	PA14_50410	Flagellar biosynthesis	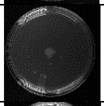	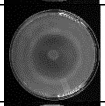
43	*flgE*	PA14_50450	Flagellar biosynthesis	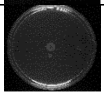	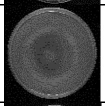
44	*flgF*	PA14_50440	Flagellar biosynthesis	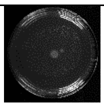	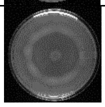
45	*flgM*	PA14_20730	Flagellar biosynthesis	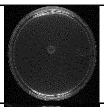	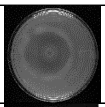
46	*flgG*	PA14_50430	Flagellar biosynthesis	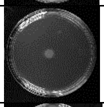	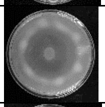
47	*fliJ*	PA14_50080	Flagellar biosynthesis	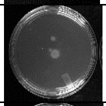	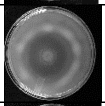
48	*flgK*	PA14_50360	Flagellar biosynthesis	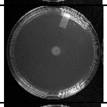	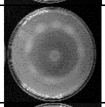
49	*fliA*	PA14_45630	Flagellar biosynthesis	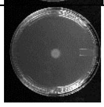	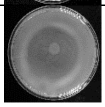
50	*flgJ*	PA14_50380	Flagellar biosynthesis	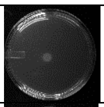	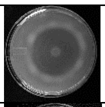
51	*hcnA*	PA14_36330	Hydrogen cyanide biosynthesis	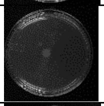	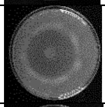
52	*hcnC*	PA14_36310	Hydrogen cyanide biosynthesis	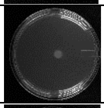	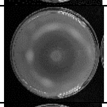
53	*pchF*	PA14_09280	Pyochelin metabolism	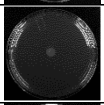	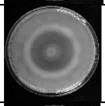
54	*pchD*	PA14_09240	Pyochelin metabolism	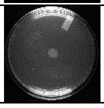	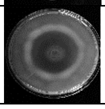

55	*pchA*	PA14_09210	Pyochelin metabolism	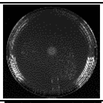	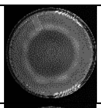
56	*pchR*	PA14_09260	Pyochelin metabolism	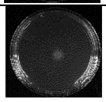	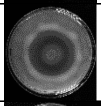
57	*pchE*	PA14_09270	Pyochelin metabolism	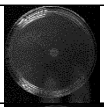	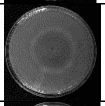
58	*fptA*	PA14_09340	Pyochelin metabolism	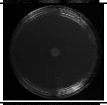	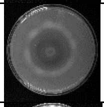
59	*pchC*	PA14_09230	Pyochelin metabolism	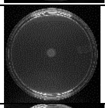	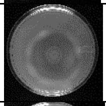
60	*pchB*	PA14_09220	Pyochelin metabolism	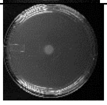	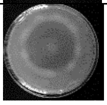
61	*pvdL*	PA14_33280	Pyoverdin metabolism	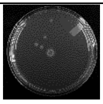	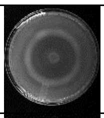
62	*pvdH*	PA14_33500	Pyoverdin metabolism	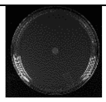	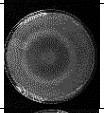
63	*pvdE*	PA14_33690	Pyoverdin metabolism	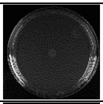	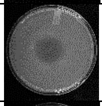
64	*pvdD*	PA14_33650	Pyoverdin metabolism	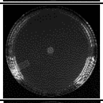	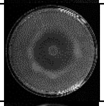
65	*pvdH*	PA14_33500	Pyoverdin metabolism	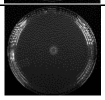	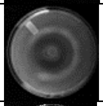
66	*pvdQ*	PA14_33820	Pyoverdin metabolism	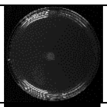	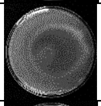
67	*pvdD*	PA14_33650	Pyoverdin metabolism	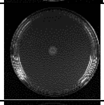	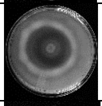
68	*pvdA*	PA14_33810	Pyoverdin metabolism	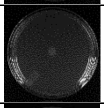	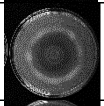
69	*pvdP*	PA14_33740	Pyoverdin metabolism	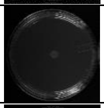	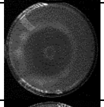
70	*pvdS*	PA14_33260	Pyoverdin metabolism	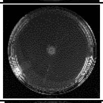	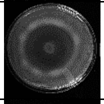
71	*fpvR*	PA14_33780	Pyoverdin metabolism	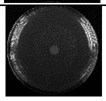	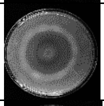
72	*fpvC*	PA14_33560	Pyoverdin metabolism	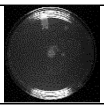	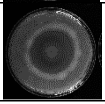

73	*pvdG*	PA14_33270	Pyoverdin metabolism	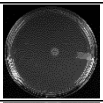	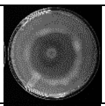
74	*fpvA*	PA14_33680	Pyoverdin metabolism	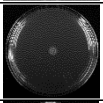	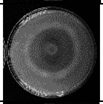
75	*pvdJ*	PA14_33630	Pyoverdin metabolism	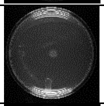	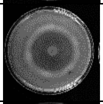
76	*wzz*	PA14_23360	LPS	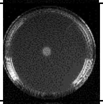	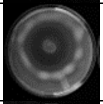
77	*pilY1*	PA14_60310	Pili	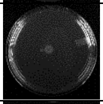	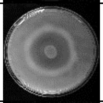
78	*pilO*	PA14_66640	Pili	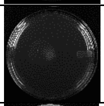	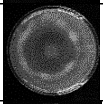
79	*pilF*	PA14_14850	Pili	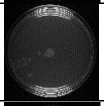	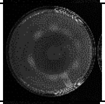
80	*cupD2*	PA14_59720	Pili	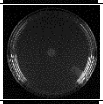	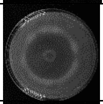
81	*pilZ*	PA14_25770	Pili	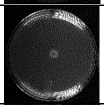	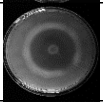
82	*pilC*	PA14_58760	Pili	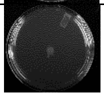	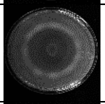
83	*chpA*	PA14_05390	Pili	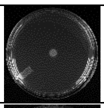	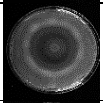
84	*pilO2*	PA14_59270	Pili	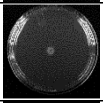	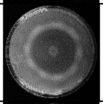
85	*pilR*	PA14_05190	Pili	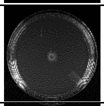	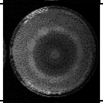
86	pili assembly chaperone	PA14_61530	Pili	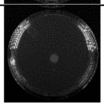	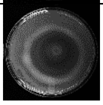
87	*pilQ2*	PA14_59290	Pili	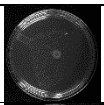	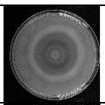
88	*fimV*	PA14_23830	Pili	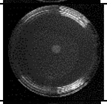	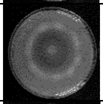
89	*pilN2*	PA14_59250	Pili	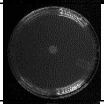	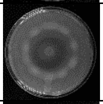
90	*fimU*	PA14_60280	Pili	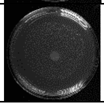	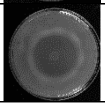

91	*pilD*	PA14_58770	Pili	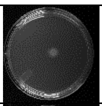	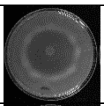
92	*pilI*	PA14_05340	Pili	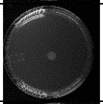	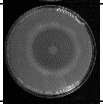
93	*cupD5*	PA14_59760	Pili	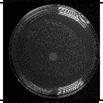	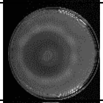
94	*pilV2*	PA14_59350	Pili	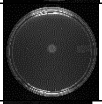	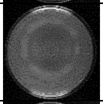
95	Tfp pilus assembly protein FimV	PA14_20860	Pili	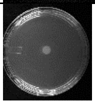	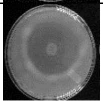
96	*fimX*	PA14_65540	Pili	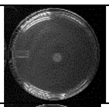	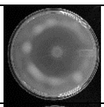
97	*pilR2*	PA14_59310	Pili	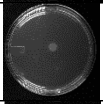	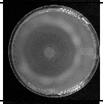
98	*aprA*	PA14_48060	Alkaline protease	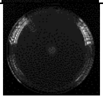	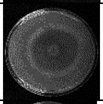
99	*LasB*	PA14_16250	LasB protease	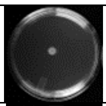	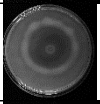
100	*LasA*	PA14_40290	Serine protease	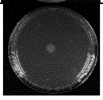	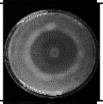
101	PhzF family phenazine biosynthesis protein	PA14_28280	Phenazine biosynthesis	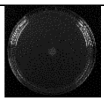	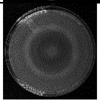
102	*phzB1*	PA14_09470	Phenazine biosynthesis	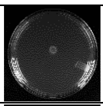	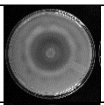
103	*phzE1*	PA14_09440	Phenazine biosynthesis	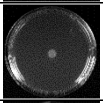	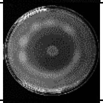
104	*phzA2*	PA14_39970	Phenazine biosynthesis	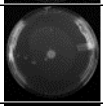	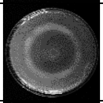
105	*phzC1*	PA14_09460	Phenazine biosynthesis	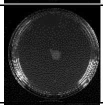	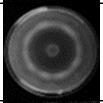
106	*phzA1*	PA14_09480	Phenazine biosynthesis	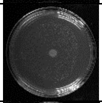	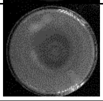
107	*phzH*	PA14_00640	Phenazine biosynthesis	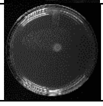	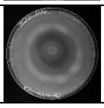

108	*phzM*	PA14_09490	Phenazine biosynthesis	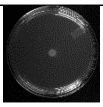	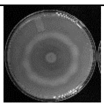
109	PhzF family phenazine biosynthesis protein	PA14_18020	Phenazine biosynthesis	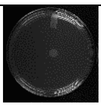	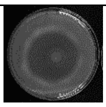
110	*phzS*	PA14_09400	Phenazine biosynthesis	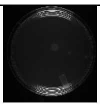	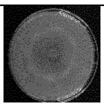
111	*phzG1*	PA14_09410	Phenazine biosynthesis	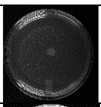	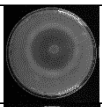
112	*phzB2*	PA14_39960	Phenazine biosynthesis	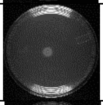	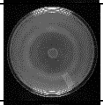
113	*rhlI*	PA14_19130	Rhl quorum sensing	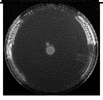	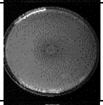
114	*rhlR*	PA14_19120	Rhl quorum sensing	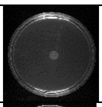	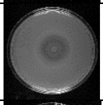
115	*lasI*	PA14_45940	Las quorum sensing	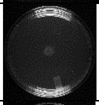	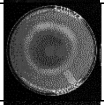
116	*lasR*	PA14_45960	Las quorum sensing	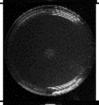	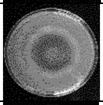
117	*pqsA*	PA14_51430	PQS signalling	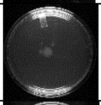	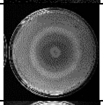
118	*pqsB*	PA14_51420	PQS signalling	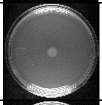	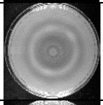
119	*pqsC*	PA14_51410	PQS signalling	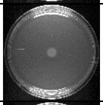	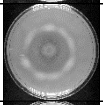
120	*pqsD*	PA14_51390	PQS signalling	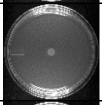	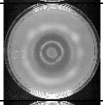
121	*pqsE*	PA14_51380	PQS signalling	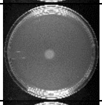	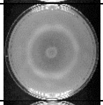
122	*pqsF*	PA14_30630	PQS signalling	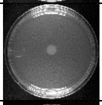	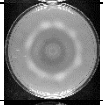
123	*eagT6*	PA14_01150	Type VI secretion system	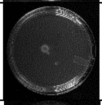	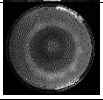
124	*tse6*	PA14_01140	Type VI secretion system	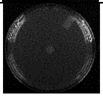	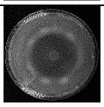

125	*pppA*	PA14_00890	Type VI secretion system	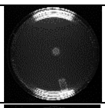	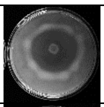
126	*tssF1*	PA14_01070	Type VI secretion system	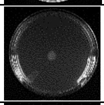	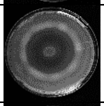
127	*hcp1*	PA14_01030	Type VI secretion system	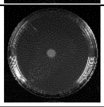	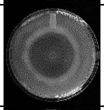
128	*tssG1*	PA14_01080	Type VI secretion system	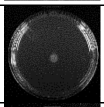	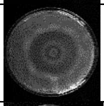
129	*tagR1*	PA14_00830	Type VI secretion system	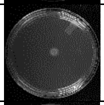	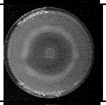
130	*vgrG1a*	PA14_01110	Type VI secretion system	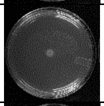	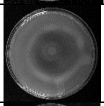
131	*tagQ1*	PA14_00820	Type VI secretion system	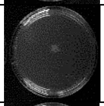	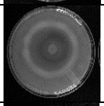
132	*tagS1*	PA14_00850	Type VI secretion system	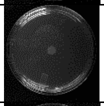	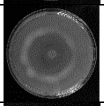
133	*hsiB1*	PA14_01010	Type VI secretion system	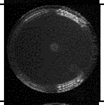	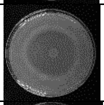
134	*tssK1*	PA14_00940	Type VI secretion system	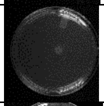	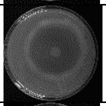
135	*tssE1*	PA14_01060	Type VI secretion system	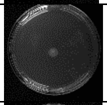	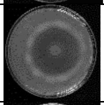
136	*hsiC1*	PA14_01020	Type VI secretion system	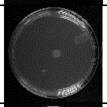	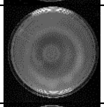
137	*icmF1*	PA14_00910	Type VI secretion system	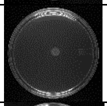	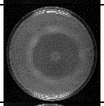
138	*clpV1*	PA14_01100	Type VI secretion system	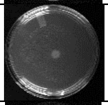	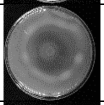
139	*tsi6*	PA14_01120	Type VI secretion system	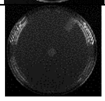	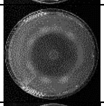
140	*fha1*	PA14_00980	Type VI secretion system	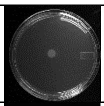	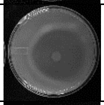
141	*hsiA2*	PA14_43050	Type VI secretion system	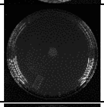	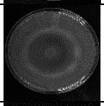
142	*fha2*	PA14_42950	Type VI secretion system	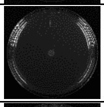	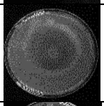
143	*stp1*	PA14_42890	Type VI secretion system	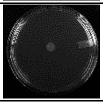	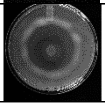

144	*sfa2*	PA14_42970	Type VI secretion system	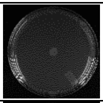	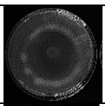
145	*dotU2*	PA14_42910	Type VI secretion system	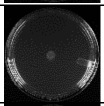	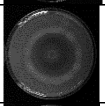
146	*stk1*	PA14_42880	Type VI secretion system	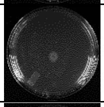	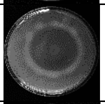
147	*clpV2*	PA14_42980	Type VI secretion system	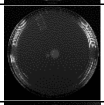	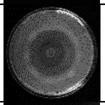
148	*hsiG2*	PA14_43000	Type VI secretion system	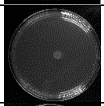	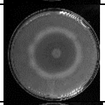
149	*hcp2*	PA14_43070	Type VI secretion system	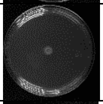	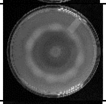
150	*tap*	PA14_43090	Type VI secretion system	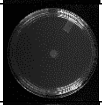	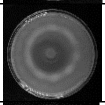
151	*icmF2*	PA14_42900	Type VI secretion system	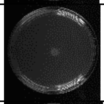	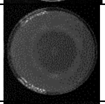
152	*hsiJ2*	PA14_42920	Type VI secretion system	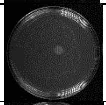	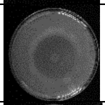
153	*lip2.1*	PA14_42940	Type VI secretion system	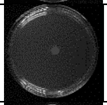	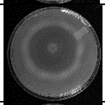
154	*hsiC2*	PA14_43030	Type VI secretion system	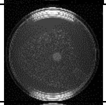	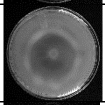
155	*rhsP2*	PA14_43100	Type VI secretion system	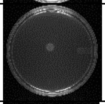	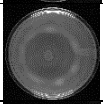
156	*lip3*	PA14_34080	Type VI secretion system	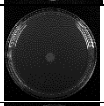	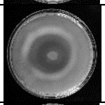
157	*hsiG3*	PA14_34010	Type VI secretion system	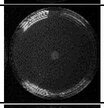	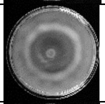
158	NA	PA14_33980	Type VI secretion system	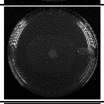	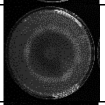
159	*icmF3*	PA14_34130	Type VI secretion system	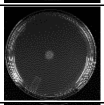	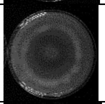
160	*hsiC3*	PA14_34050	Type VI secretion system	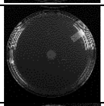	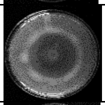
161	*dotU3*	PA14_34110	Type VI secretion system	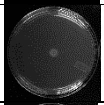	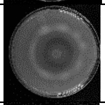
162	*sfa3*	PA14_34150	Type VI secretion system	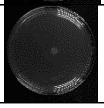	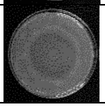

163	NA	PA14_33970	Type VI secretion system	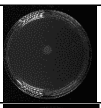	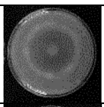
164	NA	PA14_01130	Type VI secretion system	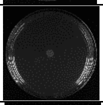	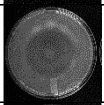
165	*xcpT*	PA14_24020	Type II secretion system	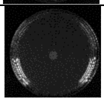	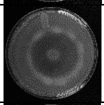
166	*xcpQ*	PA14_23970	Type II secretion system	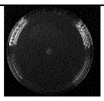	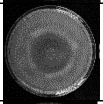
167	*hxcR*	PA14_55440	Type II secretion system	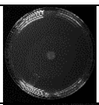	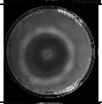
168	*xcpX*	PA14_24070	Type II secretion system	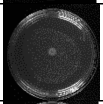	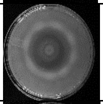
169	*xcpW*	PA14_24060	Type II secretion system	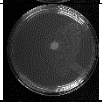	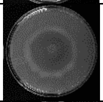
170	*xcpZ*	PA14_24100	Type II secretion system	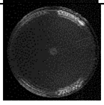	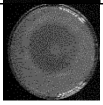
171	*hxcU*	PA14_55520	Type II secretion system	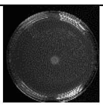	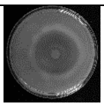
172	*xcpR*	PA14_23990	Type II secretion system	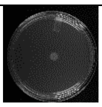	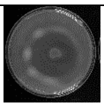
173	*exsA*	PA14_42390	Type III secretion system	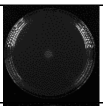	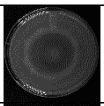
174	*pcrV*	PA14_42470	Type III secretion system	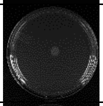	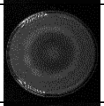
175	*pcrD*	PA14_42500	Type III secretion system	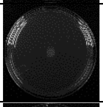	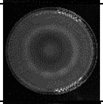
176	*pscC*	PA14_42350	Type III secretion system	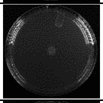	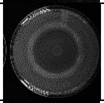
177	*pscK*	PA14_42260	Type III secretion system	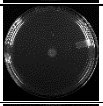	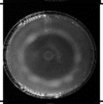
178	*popB*	PA14_42450	Type III secretion system	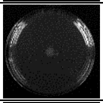	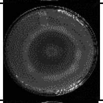
179	*pcr3*	PA14_42520	Type III secretion system	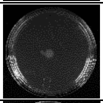	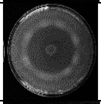
180	*pscD*	PA14_42340	Type III secretion system	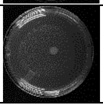	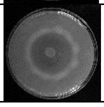

181	*pscL*	PA14_42250	Type III secretion system	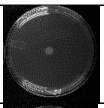	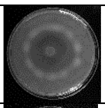
182	*popD*	PA14_42440	Type III secretion system	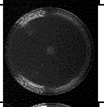	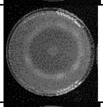
183	*pscJ*	PA14_42270	Type III secretion system	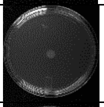	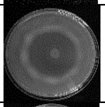
184	*pcrH*	PA14_42460	Type III secretion system	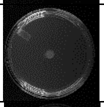	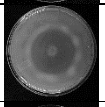
185	*CdpR*	PA14_30620	Virulence Factor Regulator	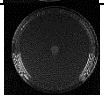	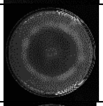
186	*GbdR*	PA14_15150	Virulence Factor Regulator	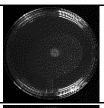	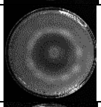
187	*AmrZ*	PA14_20290	Virulence Factor Regulator	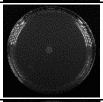	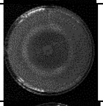
188	*SphR*	PA14_70290	Virulence Factor Regulator	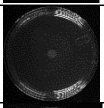	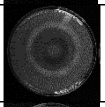
189	*QscR*	PA14_39980	Virulence Factor Regulator	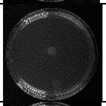	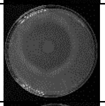
190	*MexT*	PA14_32410	Virulence Factor Regulator	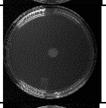	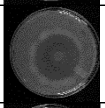
191	*MvfR*	PA14_51340	Virulence Factor Regulator	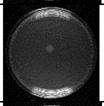	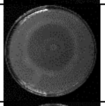
192	*RsaL*	PA14_45950	Virulence Factor Regulator	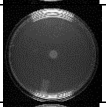	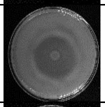
193	*VqsR*	PA14_30580	Virulence Factor Regulator	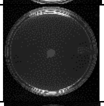	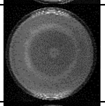
194	*toxA*	PA14_49560	Exotoxin A	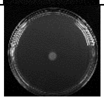	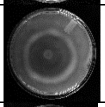
195	*exsB*	PA14_42400	Exoenzyme S synthesis protein	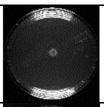	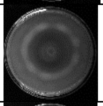
196	*exoT*	PA14_00560	Exoenzyme T	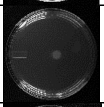	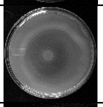
197	*exoU*	PA14_51530	Exoenzyme U	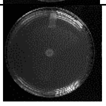	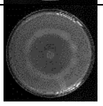
198	*excC*	PA14_42430	exsC	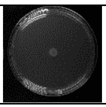	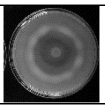

199	*plcH*	PA14_53360	Hemolytic phospholipase C	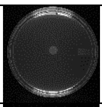	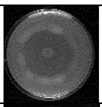
200	*plcN*	PA14_21110	Non-hemolytic phospholipase C	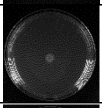	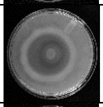
201	*plcR*	PA14_53370	Phospholipase accessory protein PlcR	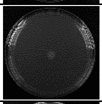	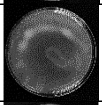
202	*plcB*	PA14_00300	Phospholipase C, PlcB	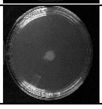	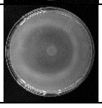
