# Supplementary material for: Toroidal displacement of Klebsiella pneumoniae by Pseudomonas aeruginosa is a unique mechanism to avoid competition for iron
Source: mBio. 2025 Jun 11;16(7):e01149-25. doi: 10.1128/mbio.01149-25 (PMC12239573; doi:10.1128/mbio.01149-25)

Toroidal displacement of *Klebsiella pneumoniae* by *Pseudomonas aeruginosa* is a unique mechanism to avoid competition for iron

Diana Pradhan, Ajay Tanwar, Joshua Wong, Srividhya Parthasarathi, Gad Frankel, Varsha Singh

Lead author: Vsingh001@dundee.ac.uk

Supplementary figures, S1 to S11

Figure S1

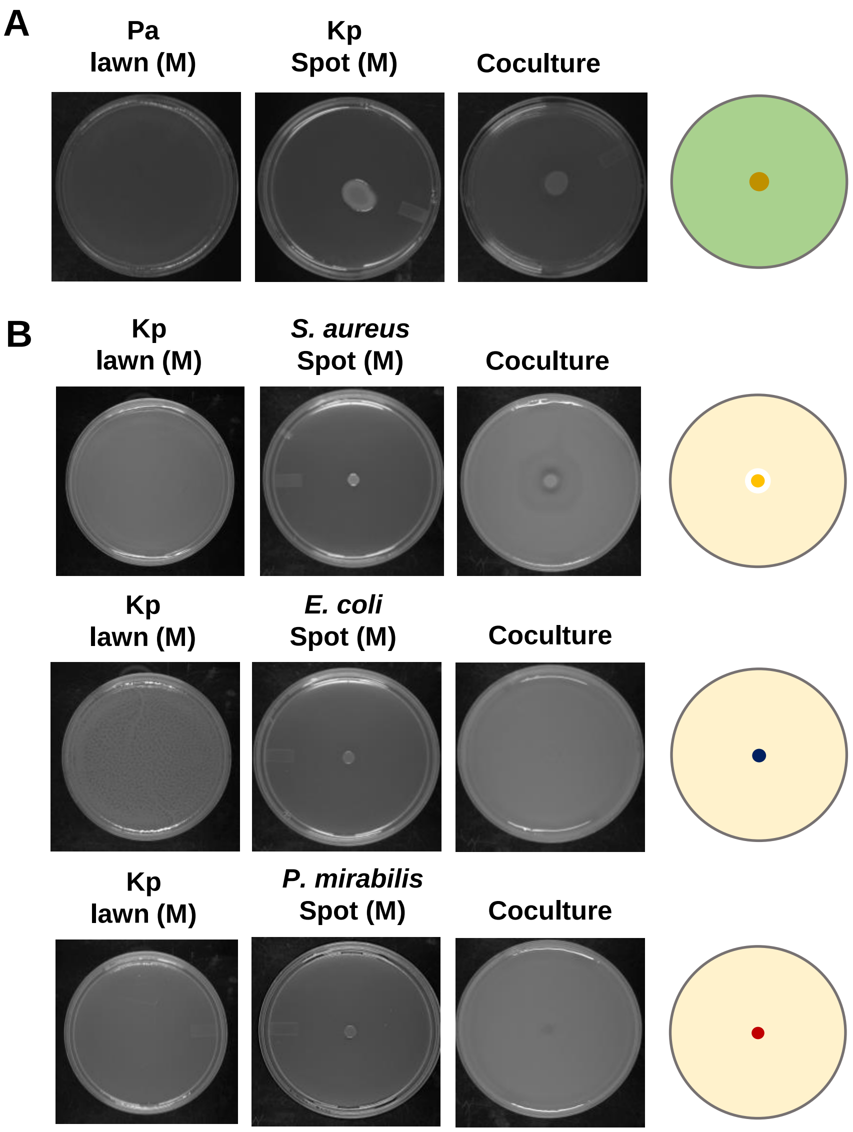
A
Pa
lawn (M)
Kp
Spot (M)
Coculture
B
Kp
lawn (M)
S. aureus
Spot (M)
Coculture
Kp
lawn (M)
E. coli
Spot (M)
Coculture
Kp
lawn (M)
P. mirabilis
Spot (M)
Coculture

Figure S2

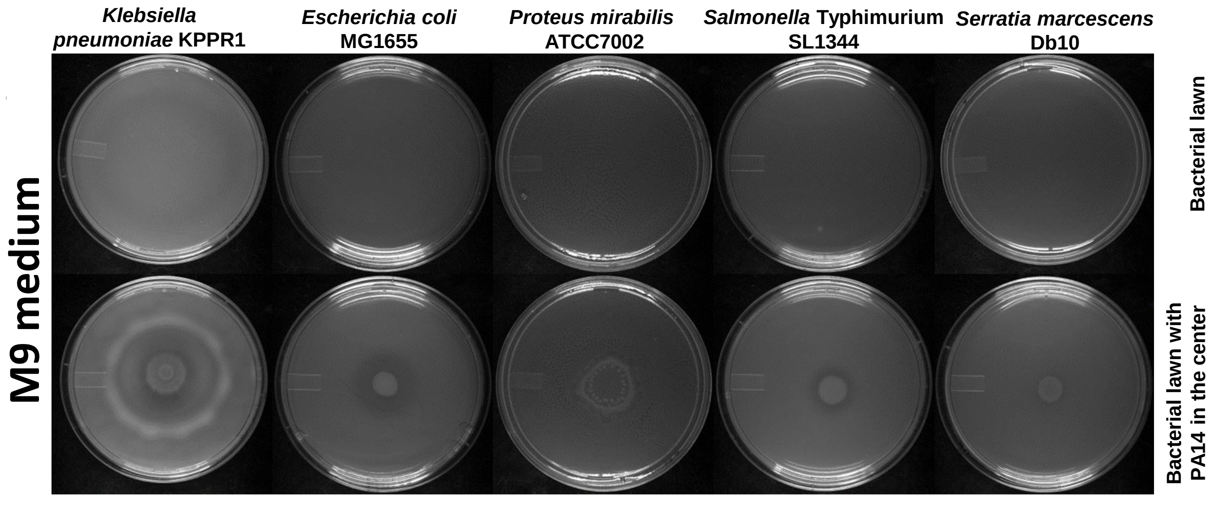

Klebsiella pneumoniae KPPR1
Escherichia coli MG1655
Proteus mirabilis ATCC7002
Salmonella Typhimurium SL1344
Serratia marcescens Db10
M9 medium
Bacterial lawn
Bacterial lawn with PA14 in the center

Figure S3

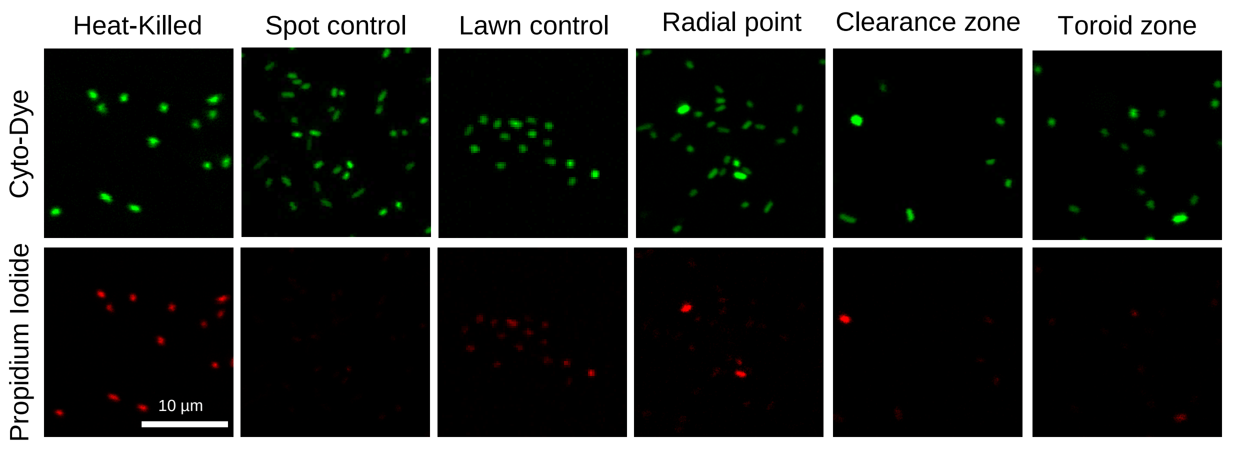
Heat-Killed
Spot control
Lawn control
Radial point
Clearance zone
Toroid zone
Cyto-Dye
Propidium Iodide
10 µm

Figure S4

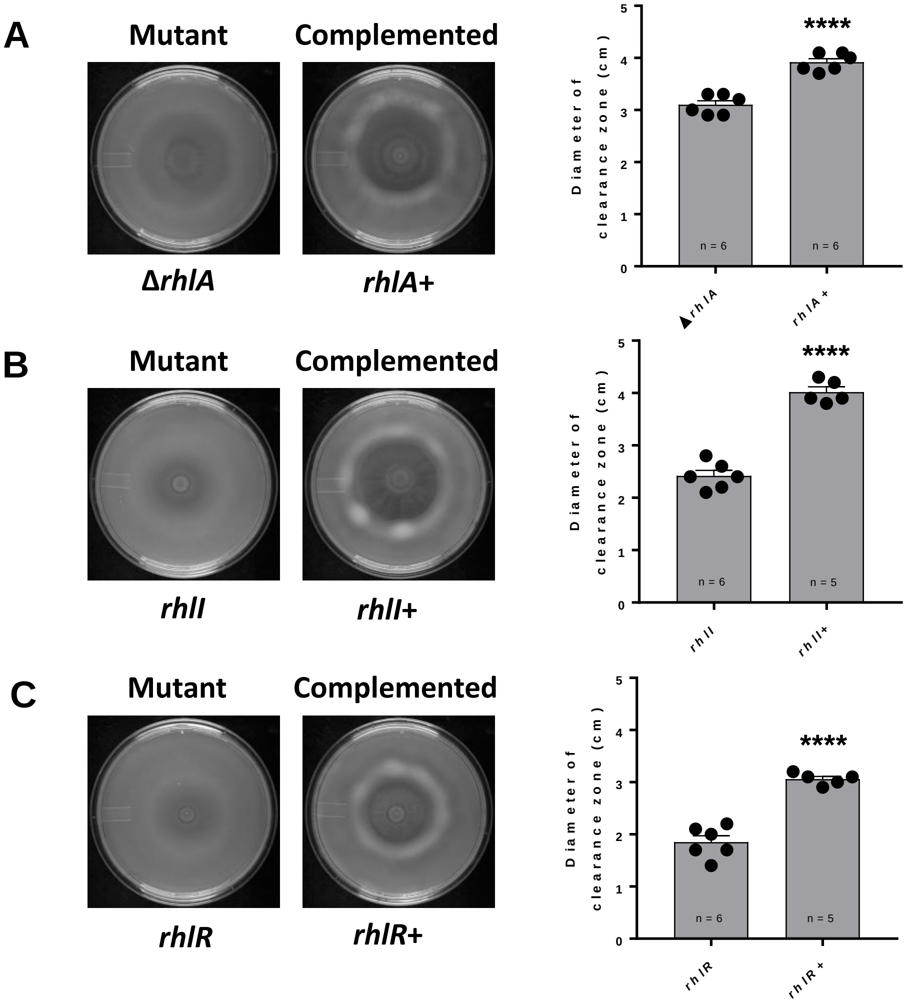
A
Mutant
Complemented
ΔrhlA
rhlA+
Diameter of clearance zone (cm)
****
n = 6
n = 6
ΔrhlA
rhlA+
B
Mutant
Complemented
rhlI
rhlI+
Diameter of clearance zone (cm)
****
n = 6
n = 5
rhlI
rhlI+
C
Mutant
Complemented
rhlR
rhlR+
Diameter of clearance zone (cm)
****
n = 6
n = 5
rhlR
rhlR+

Figure S5

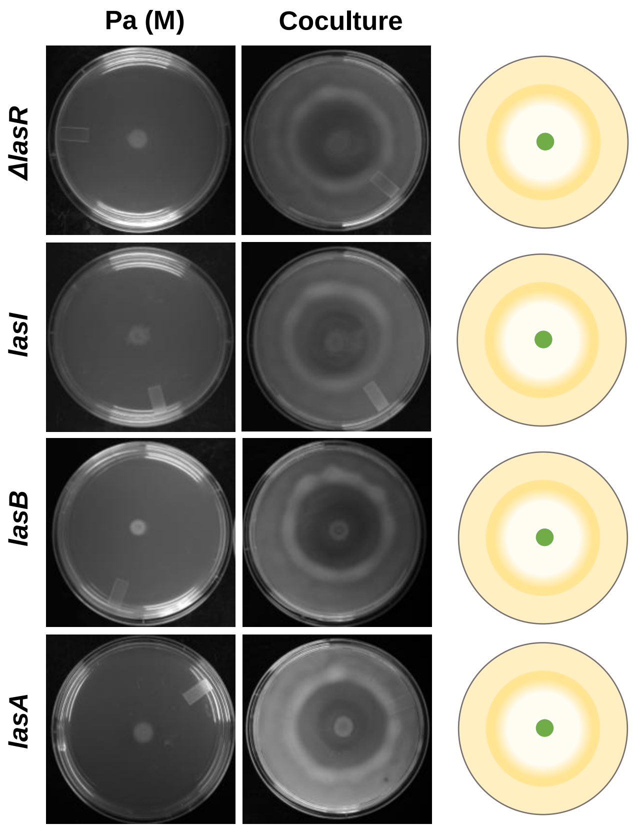

Figure S6

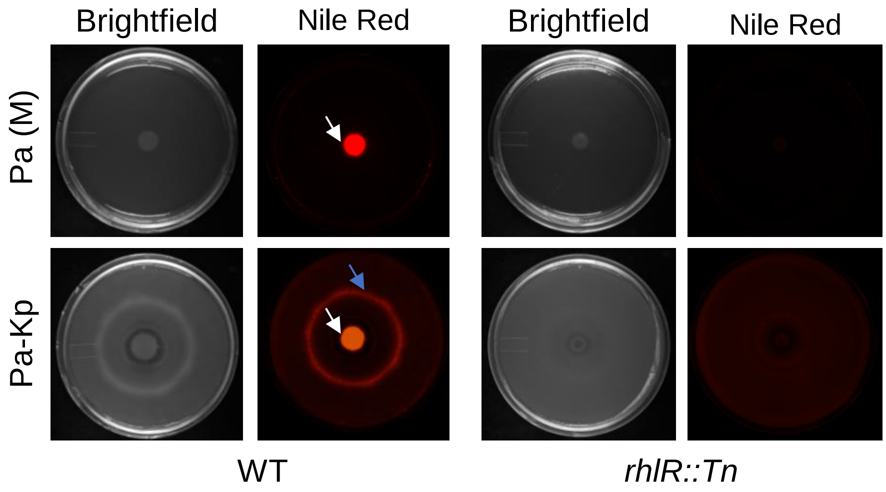
Brightfield
Nile Red
Brightfield
Nile Red
Pa (M)
Pa-Kp
WT
rhlR::Tn

Figure S7

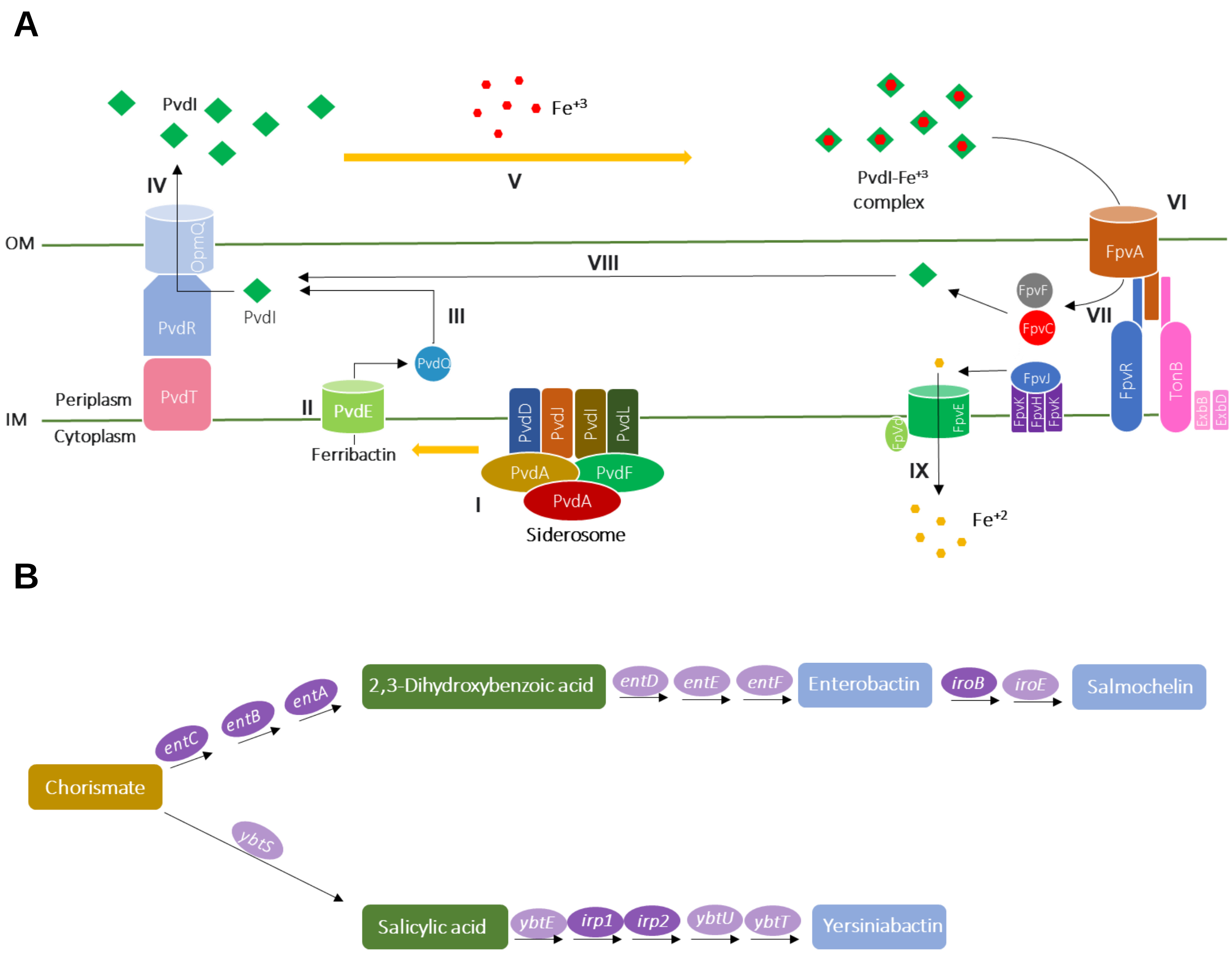
A
PvdI
Fe^{+3}
V
PvdI-Fe^{+3} complex
VI
IV
OM
OpmQ
VIII
FpvA
PvdR
PvdI
III
FpvF
VII
FpvC
PvdQ
FpvR
TonB
FpvJ
Periplasm
PvdT
II
PvdE
PvdD
PvdJ
PvdI
PvdL
FpvE
FpvK
FpvH
FpvK
ExbB
ExbD
IM
Cytoplasm
Ferribactin
FpvG
PvdA
PvdF
IX
I
PvdA
Siderosome
Fe^{+2}
B
2,3-Dihydroxybenzoic acid
entD
entE
entF
Enterobactin
iroB
iroE
Salmochelin
entA
entB
entC
Chorismate
ybtS
Salicylic acid
ybtE
irp1
irp2
ybtU
ybtT
Yersiniabactin

Figure S8

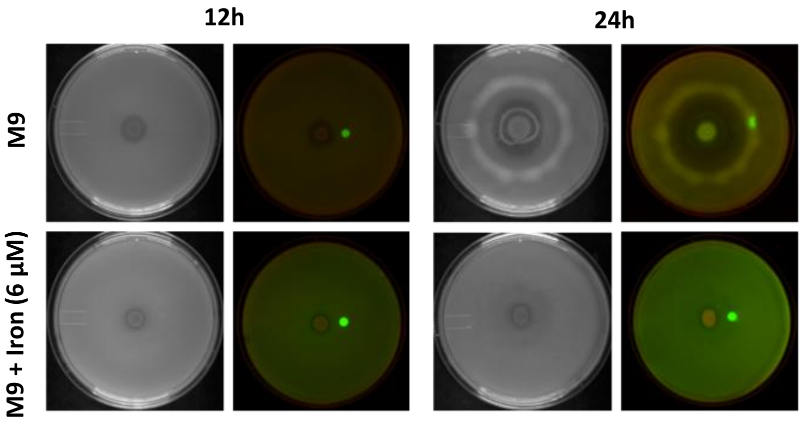

Figure S9

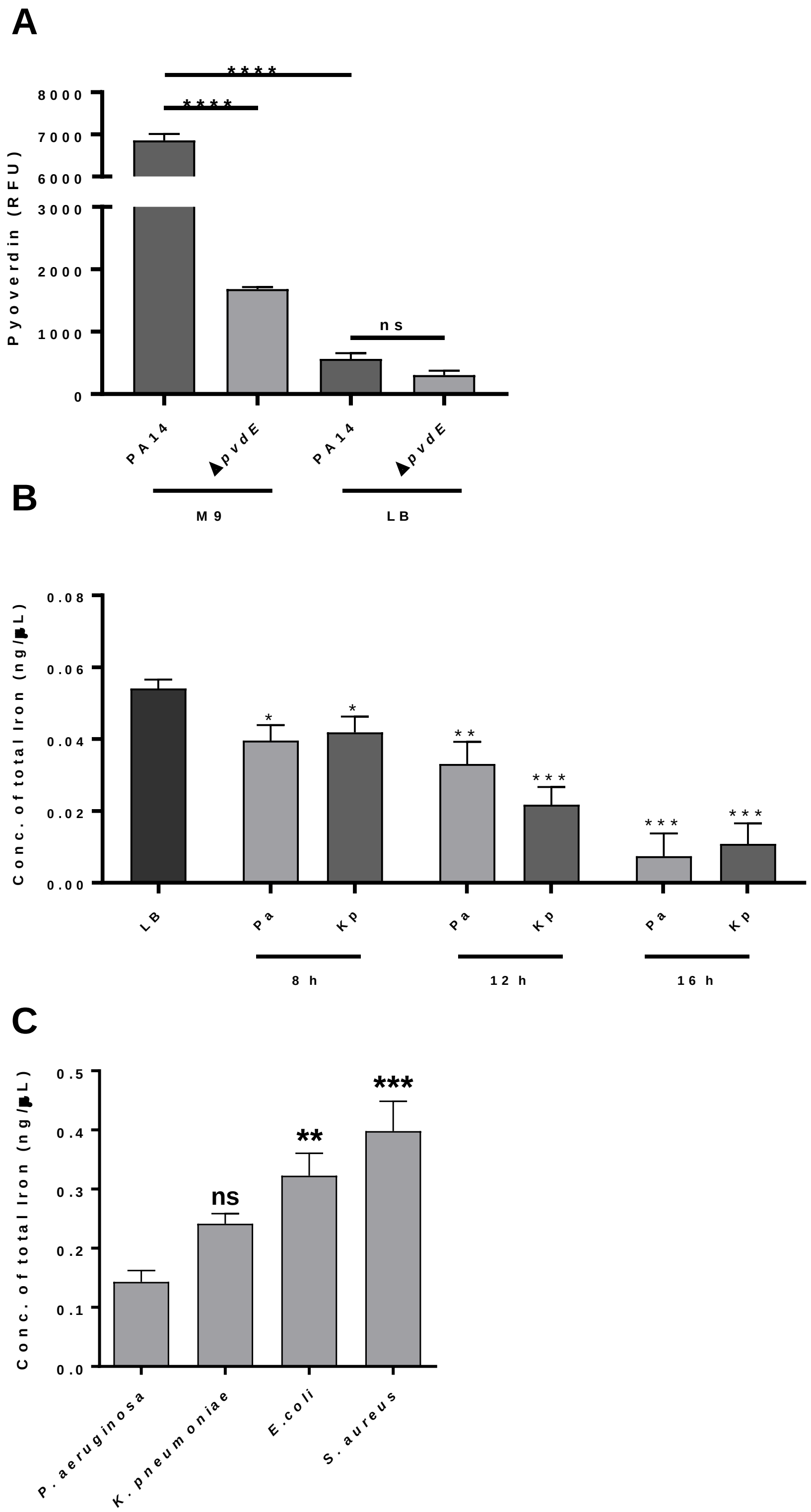
A
B
C
Pyoverdin (RFU)
8000
7000
6000
3000
2000
1000
0
****
****
ns
PA14
ΔpvdE
PA14
ΔpvdE
M9
LB
Conc. of total Iron (ng/μL)
0.08
0.06
0.04
0.02
0.00
*
*
**
***
***
***
LB
Pa
Kp
Pa
Kp
Pa
Kp
8 h
12 h
16 h
Conc. of total Iron (ng/μL)
0.5
0.4
0.3
0.2
0.1
0.0
ns
**
***
P. aeruginosa
K. pneumoniae
E. coli
S. aureus

Figure S10

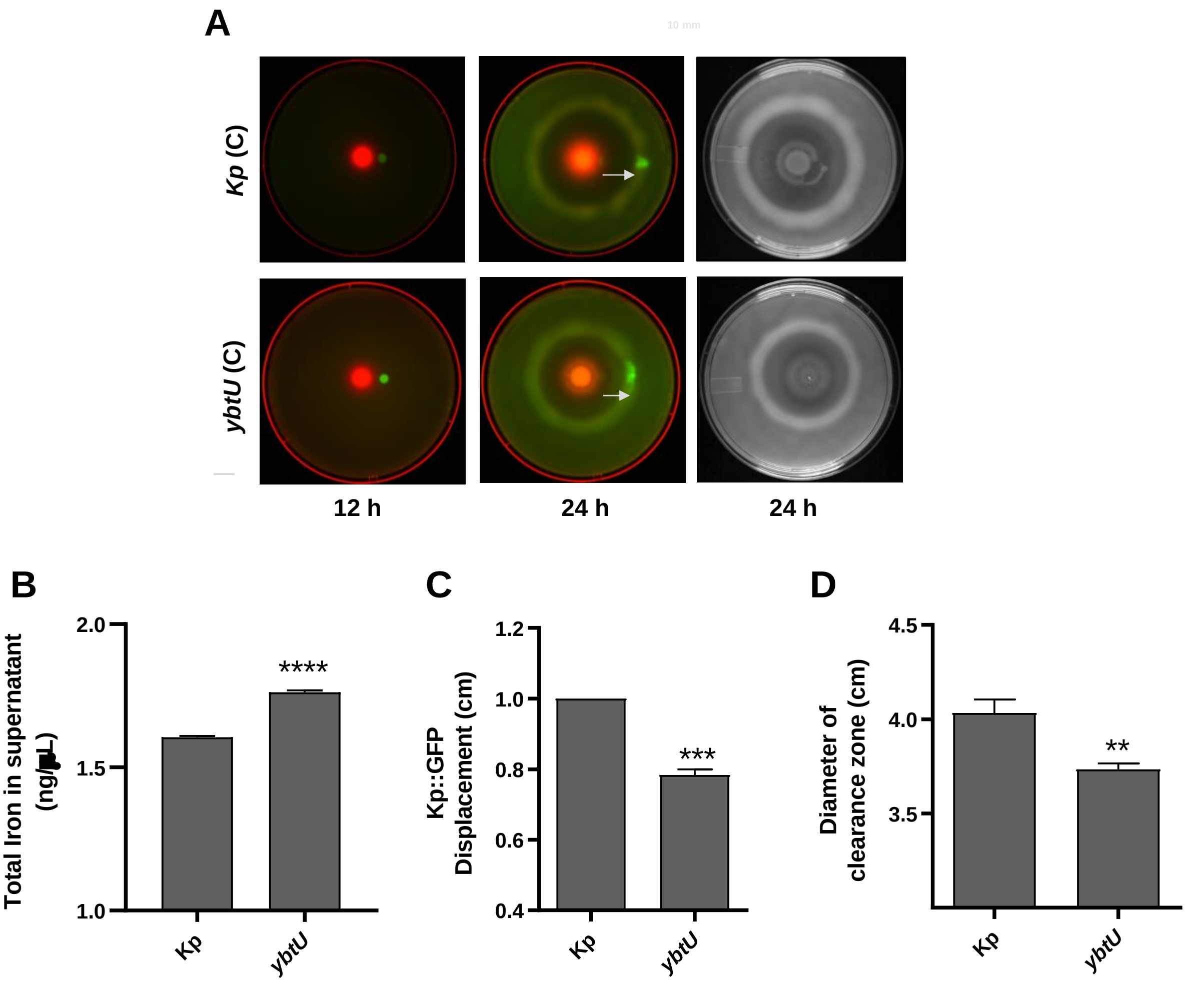

A
10 mm
Kp (C)
ybtU (C)
12 h
24 h
24 h
B
Total Iron in supernatant (ng/µL)
2.0
1.5
1.0
****
Kp
ybtU
C
Kp::GFP Displacement (cm)
1.2
1.0
0.8
0.6
0.4
***
Kp
ybtU
D
Diameter of clearance zone (cm)
4.5
4.0
3.5
**
Kp
ybtU

Figure S11

A

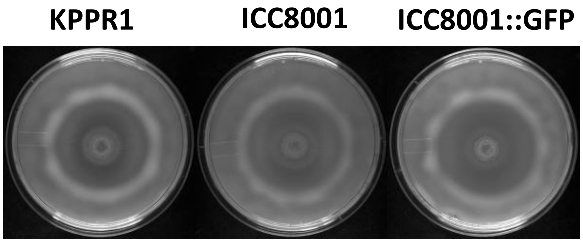

B

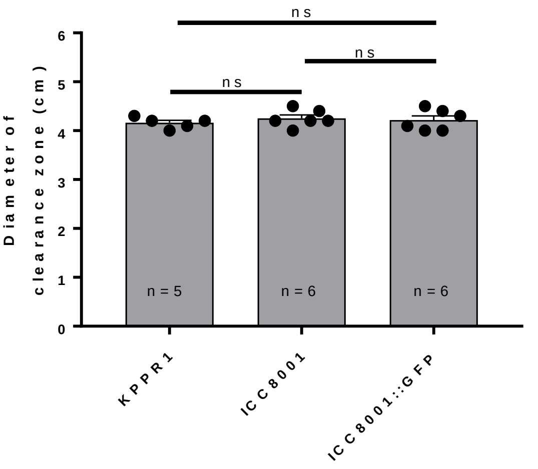

Supplement: Supplemental Figures — Figure S1 to S11. [file mbio.01149-25-s0006.pdf]
